# Circulating Tumor DNA as a Biomarker for Precision Medicine in Prostate Cancer: A Systematic Review

**DOI:** 10.3390/ijms262211049

**Published:** 2025-11-15

**Authors:** Nouhaila Chanhih, Abdelilah Laraqui, Salma Hassine, Ahmed Ameur, Larbi Hamedoun, Hicham El Annaz, Rachid Abi, Mohamed Rida Tagajdid, Idriss Lahlou Amine, Khalid Ennibi, Abdelaziz Benjouad, Lamiae Belayachi

**Affiliations:** 1 Center for Research on Health Sciences (CReSS), International Faculty of Medicine, College of Health Sciences, International University of Rabat, Technopolis Parc, Rocade of Rabat-Salé, Sala-Al Jadida 11100, Morocco; nouhaila.chanhih@uir.ac.ma (N.C.); abdelaziz.benjouad@uir.ac.ma (A.B.); 2 Laboratory of Virology, Center of Virology, Infectious and Tropical Diseases, Mohammed V Military Teaching Hospital, Faculty of Medicine and Pharmacy, Mohammed V University, Rabat 10045, Morocco; loranjad@yahoo.fr (A.L.); hichameelannaz74@gmail.com (H.E.A.); aabirachid@gmail.com (R.A.); tagajdid@gmail.com (M.R.T.); idr_lahlou@yahoo.com (I.L.A.); 3 Biomedical and Epidemiological Research Unit, Center of Virology, Infectious and Tropical Diseases, Mohammed V Military Teaching Hospital, Faculty of Medicine and Pharmacy, Mohammed V University, Rabat 10045, Morocco; salma-hassine@um5r.ac.ma; 4 Laboratory of Biology and Human Pathologies, Department of Biology, Faculty of Sciences of Rabat, University Mohammed V of Rabat, Rabat 10000, Morocco; 5 Department of Urology, Mohammed V Military Teaching Hospital, Faculty of Medicine and Pharmacy, Mohammed V University, Rabat 10045, Morocco; ameur@yahoo.com (A.A.); larbi771@gmail.com (L.H.); 6 Center of Virology, Infectious and Tropical Diseases, Mohammed V Military Teaching Hospital, Faculty of Medicine and Pharmacy, Mohammed V University, Rabat 10045, Morocco; kennibi@yahoo.fr

**Keywords:** precision medicine, precision oncology, prostatic neoplasms, circulating tumor DNA, cancer biomarkers, liquid biopsy

## Abstract

Circulating tumor DNA (ctDNA) profiling offers non-invasive insights for personalized prostate cancer management. This systematic review provides the first comprehensive appraisal of ctDNA assay methods, genomic targets, and their clinical correlations and proposes practical recommendations to guide future standardization and validation. We searched PubMed, ScienceDirect, Scopus, and the Cochrane Library starting December 2024 following PRISMA (Preferred Reporting Items for Systematic reviews and Meta-Analyses) guidelines. From 229 records, 44 studies (10,631 patients) met the inclusion criteria. Plasma ctDNA analyzed by NGS predominantly profiled TP53 (72.7%), AR (70.4%), BRCA1/2 (61.3%), ATM (50%), RB1 (47.7%), and PTEN (41%). ctDNA positivity and specific key alterations correlated with poorer overall and progression-free survival. BRCA1/2-mutant patients benefited from Olaparib plus Abiraterone, while persistent alterations predicted early progression. Beyond synthesizing existing evidence, we identify key gaps, such as inconsistent reporting of variant allele fractions, limited diversity in study populations, and underexplored rare alterations. We recommend unified reporting standards (e.g., variant allele frequency thresholds and panel composition) and prioritized prospective trials to validate high-impact targets. These steps will accelerate the integration of ctDNA into routine precision oncology practice worldwide.

## 1. Introduction

Precision medicine (PM) in oncology is an emerging approach that relies on distinct characteristics of a sub-population, such as genetic profile, environment, and lifestyle, to predict disease risk, diagnose disease, tailor therapies, and reduce clinical complications. This approach advances cancer care by shifting from a one-size-fits-all paradigm to molecularly guided interventions. In prostate cancer (PCa), PM tools such as OMICs have shown promise in prevention, diagnosis, prognosis, and therapies, offering increased efficacy and reduced toxicity [1].

PCa is the second most commonly diagnosed cancer among men and one of the leading causes of cancer-related mortality worldwide [2]. It is a highly heterogeneous disease, with clinical behaviors ranging from indolent to highly lethal and genetic backgrounds that influence prognosis and therapeutic decisions in advanced stages. Traditional diagnostic approaches rely on prostate-specific antigen (PSA) blood testing, digital rectal examination (DRE), and confirmation through transrectal ultrasound (TRUS)-guided biopsy. However, both PSA testing and DRE have limited specificity and sensitivity: DRE does not improve PSA-based detection [3], and PSA testing can be influenced by noncancerous conditions such as benign prostatic hyperplasia or inflammation [4], as well as by age.

Liquid biopsy has emerged as a promising alternative, providing a dynamic and non-invasive tool to characterize tumor biology and support the realization of PM [5]. It can guide the need for tissue biopsy, offer prognostic insights in advanced disease, and monitor treatment response in clinical trials. Nonetheless, its integration into clinical workflows remains limited [6].

Liquid biopsy detects circulating tumor-derived analytes such as ctDNA, cfDNA, exosomes, and proteins, reflecting tumor heterogeneity and evolution [7]. Among these, ctDNA is the most widely studied marker in clinical practice. Derived from apoptotic or necrotic tumor cells, ctDNA shows high sensitivity and specificity across multiple cancer types and stages [8]. Through genomic analysis, ctDNA enables early detection, determination of tissue of origin, prognosis, treatment monitoring, assessment of resistance, and detection of minimal residual disease [9].

In PCa, the ctDNA fraction correlates with overall survival (OS), progression-free survival (PFS), and treatment response, outperforming several established prognostic factors [10]. Detectable alterations from ctDNA, such as AR and HRR mutations, have been identified as predictive markers in castration-resistant PCa (CRPC) [11,12,13,14], while the ctDNA fraction increases with disease progression in hormone-sensitive PCa (HSPC) [15,16].

We therefore conducted a systematic review to investigate the relationship between genetic alterations detected through ctDNA sequencing and clinical outcomes in localized and metastatic PCa. Our aim is to highlight clinically meaningful alterations and assess their translational potential, thereby clarifying the role of ctDNA in precision medicine and its future integration into clinical practice.

## 2. Materials and Methods

**Figure 1:**
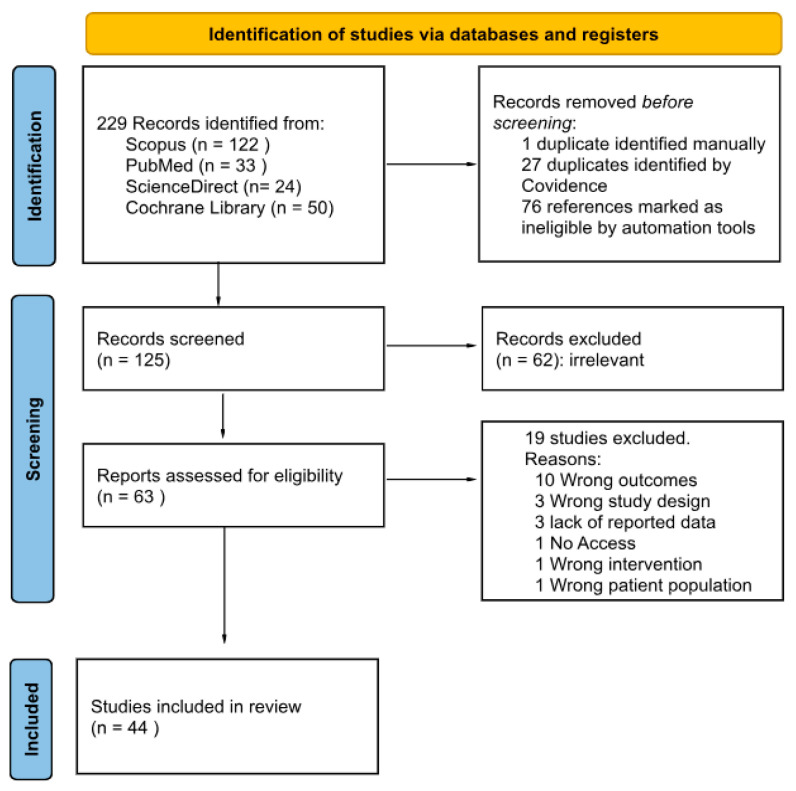
PRISMA flow diagram of study selection. This diagram illustrates the study selection process for the systematic review, based on the PRISMA 2020 guidelines. The number of records identified, screened, assessed for eligibility, and included in the final review is shown, along with reasons for exclusions at each stage.

Our review adhered to the Preferred Reporting Items for Systematic Review and Meta-analyses (PRISMA) guidelines (Figure 1). It was preregistered on the International Prospective Register of Systematic Reviews (CRD42025628570).

### 2.1. Search Strategy

A systematic literature review of PubMed, ScienceDirect, Scopus, and the Cochrane Library was conducted. These databases were selected to ensure comprehensive yet specific coverage of relevant clinical research and systematic reviews. We included prospective and retrospective peer-reviewed publications and research abstracts on ctDNA use for detection, monitoring, and prognostication of PCa and for assessment of treatment responses. Keywords included (“circulating tumor DNA” OR “ctDNA”) AND (“prostate cancer” OR “prostate carcinoma” OR “prostatic neoplasm” OR “prostate malignancy” OR “prostate tumor”) AND (“alterations” OR “mutations”) AND (“clinical significance” OR “diagnosis” OR “prognosis” OR “treatment” OR “therapy” OR “monitoring” OR “outcomes”). In order to include unpublished randomized controlled trials (RCTs) and trial updates, we also reviewed abstracts presented at recent relevant conferences between 2015 and 2024. The primary outcomes of interest were the clinical impact associated with detected genetic alterations in patients’ ctDNA such as OS, PFS, or response to treatment. Two investigators performed the initial screening based on the titles and abstracts to detect eligible studies. Potentially relevant studies were then subject to a full-text review. Disagreements were resolved by discussions with the co-authors.

Only articles published in English and peer-reviewed journals were included. To confirm the peer-reviewed status of the journals, we verified each journal’s indexing in reputable databases such as PubMed, Scopus, and the Cochrane Library. Additionally, we checked the editorial policies of the journals to ensure that they follow a peer-review process. This verification process ensured that all included studies met our criteria for scientific rigor and quality. To evaluate the quality and reliability of the included studies, we conducted a risk-of-bias assessment using the Cochrane Risk of Bias tool for randomized controlled trials, Quality In Prognostic Studies (QUIPS) for Prognostic Factor Studies, The Joanna Briggs Institute (JBI), and the Newcastle–Ottawa Scale (NOS) for observational studies. Each study was independently assessed by two reviewers, and discrepancies were resolved through discussion. Identified risks of bias were discussed in the Results and Recommendations sections to provide a transparent evaluation of the potential impact on the review’s findings.

We also searched for pertinent abstracts from leading multidisciplinary medical oncology associations, including the American Society of Clinical Oncology and the European Society for Medical Oncology, to uncover the latest research and newly published studies.

To address potential duplication in the data, studies with overlapping datasets were carefully evaluated and used the assistance of Covidence tools. Preference was given to the most comprehensive and recent publications, prioritizing studies based on their dataset completeness, publication date, and relevance. This approach ensured that duplicate data did not bias the findings.

To further mitigate the risk of duplication, we used The National Clinical Trial (NCT) numbers to identify publications arising from the same clinical trials. In cases where multiple publications with the same NCT number were identified, these publications were grouped, and their datasets were compared to identify overlaps. Data from the most comprehensive and recent publication were included to avoid redundancy and bias. Distinct analyses or non-overlapping data reported across publications were considered separately and appropriately documented.

Duplicate titles were first eliminated. We then conducted a title check to assess relevance. The remaining publications’ abstracts were examined, and irrelevant ones were removed. For the remaining articles, full-text manuscripts and/or conference posters or presentations were reviewed. When multiple versions of the same data were available, preference was given to the most comprehensive and recent updates.

### 2.2. Inclusion and Exclusion Criteria

Studies were selected if they investigated localized or advanced PCa patients (patients), who underwent ctDNA testing (interventions) compared to those who did not undergo ctDNA testing or who did not detect any alterations in their ctDNA (comparisons) to assess the differential pathologic clinical outcomes (outcome) in case reports, clinical trials, meta-analyses, multicenter studies, observational studies, RCTs, systematic reviews, clinical studies, cohort studies, and original research (study design). Studies lacking original patient data, letters, editorial comments, replies from authors, and non-English language papers were excluded. All publications included had their references checked for relevant additional research.

### 2.3. Data Extraction

Two authors independently extracted the following data: author(s), year of publication, study design, country, sample size, inclusion/exclusion criteria, age, disease stage (localized or advanced PCa), treatment regimen, type of ctDNA extraction and detection methods, and genetic alterations identified. Data related to OS, PFS, treatment response, and any other relevant clinical outcomes (e.g., recurrence and progression) were also extracted in addition to specific ctDNA alterations identified and associated clinical outcomes. Available hazard ratios (HRs), *p*-values, and confidence intervals (CIs) of each clinical outcome were collected.

### 2.4. Risk-of-Bias Assessment

The risk of bias was evaluated using five validated tools, each tailored to the study design of the included papers. For RCTs, the RoB 2 tool was applied to assess bias across five domains: the randomization process, deviations from intended interventions, missing outcome data, measurement of the outcome, and selection of the reported result. For prognostic studies examining associations between ctDNA features and clinical outcomes—whether standalone or embedded within larger trials—the QUIPS tool was used. This framework evaluates six domains: study participation, study attrition, prognostic factor measurement, outcome measurement, study confounding, and statistical analysis/reporting.

The NOS was used to assess the methodological quality of non-randomized cohort and observational studies, including both retrospective and prospective designs. The NOS evaluates three broad domains: selection of study groups, comparability of cohorts, and ascertainment of either the exposure or the outcome of interest. For non-randomized studies of interventions that aimed to explore causal relationships, the Risk Of Bias In Non-randomized Studies-of Interventions (ROBINS-I) tool was applied. This tool assesses the risk of bias across seven domains: confounding, selection of participants, classification of interventions, deviations from intended interventions, missing data, measurement of outcomes, and selection of the reported result. Each domain, as well as the overall study, was rated as having low, moderate, serious, or critical risk of bias.

In addition, the JBI critical appraisal checklist for analytical cross-sectional studies was used for studies with a cross-sectional design. This tool assesses methodological soundness based on inclusion criteria, measurement validity, confounding factors, and statistical analysis. Case reports, abstract-only or in vitro-only studies, and small descriptive case series were excluded from formal risk-of-bias assessment due to the lack of applicable tools and their limited generalizability.

Two authors independently performed the risk-of-bias assessments using the appropriate tool for each study type. Discrepancies were resolved through discussion and consensus.

## 3. Results and Discussion

### 3.1. Eligible Studies

A total of 229 publications were identified through database search (Figure 1). Of these, 44 (19.2%) met the inclusion criteria and were included in our systematic review.

Most studies were conducted in North America (13, 29.5%) [17,18,19,20,21,22,23,24,25,26,27,28,29] and Asia (10, 22.7%) [30,31,32,33,34,35,36,37,38,39]. Additional studies were conducted in Europe (2, 4.5%) [40,41], across multiple continents (17, 38.6%) [42,43,44,45,46,47,48,49,50,51,52,53,54,55,56,57,58], Australia (2, 4.5%) [59,60], and South America (1, 2.3%) [61]. Overall, 17 (38.6%) studies were retrospective [18,19,26,27,28,29,31,32,33,34,35,39,42,43,53,55,59], 7 (15.9%) were prospective [21,23,30,40,44,45,60], and 10 (22.7%) were RCTs [46,47,48,49,52,54,56,57,58,61]. The remaining (9, 20.5%) were case reports, case series, or observational biomarker studies [20,22,23,25,37,38,50]. One study included both prospective and retrospective cohorts [51]. The longest study period was reported by Reimers et al. [19], which covered 30 years (1988–2018), while the shortest study by Du et al. [34] lasted only 8 months (October 2019–June 2020). The majority of studies focused on data collected within the last decade, with several large trials, such as those by Oya et al. [46] and Clarke et al. [47], contributing extensive patient cohorts. Study characteristics are provided in the Supplementary Material (Table S1)).

### 3.2. Risk-of-Bias 

Risk of bias was assessed across all eligible included studies using validated tools tailored to their respective study designs. For randomized controlled trials, the risk-of-bias (RoB) 2 tool identified two studies, both with an overall risk of some concerns (Supplementary Table S2).

The QUIPS tool was applied to prognostic studies evaluating ctDNA features and clinical outcomes, with 12 out of 20 studies judged as low risk across most domains and 8 studies presenting moderate risk due to limitations in prognostic factor or outcome measurement, study participation or attrition, confounding control, or incomplete reporting (Supplementary Table S3).

Among non-randomized cohort and observational studies assessed using the NOS, two studies out of five were rated as high quality (low risk of bias), while three showed moderate quality due to weaknesses in comparability, outcome, or selection (Supplementary Table S4).

ROBINS-I was used for two non-randomized intervention studies, with one rated at moderate risk and one at serious risk of bias, largely due to confounding, missing data, and selective reporting (Supplementary Table S5). Additionally, two analytical cross-sectional studies were evaluated using the JBI tool, with one rated as serious and one showing some risk mainly due to confounding (Supplementary Table S6).

Together, these assessments ensured that the diverse study designs were evaluated using appropriate, design-specific tools. While most studies were rated as having good and moderate quality, caution is warranted when interpreting findings from non-randomized and analytical cross-sectional studies due to residual confounding, limited control over selection bias, and variable reporting standards.

### 3.3. Study and Patient Characteristics

**Figure 2:**
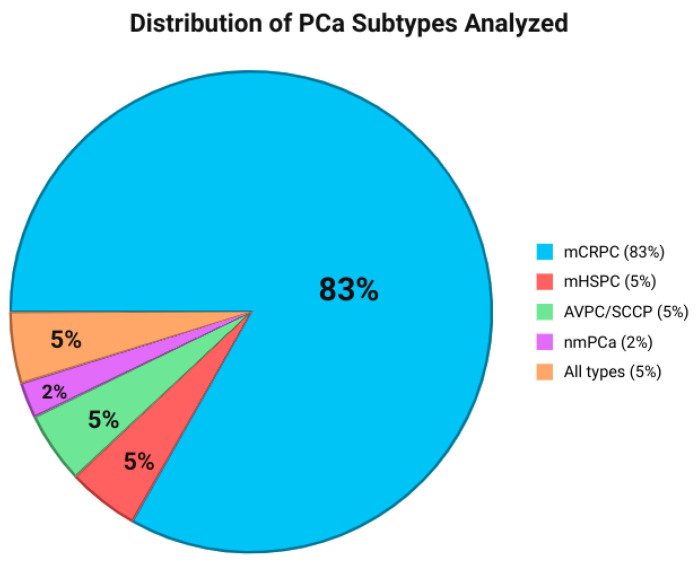
Distribution of prostate cancer subtypes analyzed in ctDNA studies. Pie chart showing the distribution of prostate cancer (PCa) subtypes investigated across studies that reported subtype information (n = 44). This distribution indicates a predominant research focus on ctDNA in mCRPC, with limited representation of earlier-stage or variant subtypes.

The study and patients’ characteristics are reported in the Supplementary Material (Table S1). Metastatic CRPC (mCRPC) was analyzed in thirty-five (79.5%) studies [18,19,20,21,22,24,26,27,29,32,33,36,37,38,40,41,42,43,45,46,47,49,50,51,52,54,55,56,57,58,59,60,61], Metastatic HSPC (mHSPC) was analyzed in two (4.5%) studies [31,34], Aggressive-Variant Prostate Cancer (AVPC)/Small Cell Carcinoma of Prostate (SCCP) was present in two (4.5%) studies [25,39], and Non-metastatic Prostate Cancer (nmPCa) was analyzed in one (2.2%) study [35]. The remaining studies did not restrict the PCa subtype in their analysis and included all PCa subtypes (2, 4.5%) [30,33] (Figure 2). OS was the most frequent outcome (21, 47.7%) [18,19,22,23,26,29,32,36,38,41,42,43,45,47,49,51,52,54,58,59,60], followed by PFS (17, 38.6%) [18,19,22,23,26,32,36,38,41,42,43,45,49,51,54,59,60], PSA Response or PSA-PFS (11, 25%) [18,19,22,23,32,36,42,43,49,51,59], rPFS (10, 22.7%) [19,22,23,36,43,45,49,51,54,59], Time To Progression (TTP) (5, 11.4%) [19,22,43,49,51], Time on Treatment (ToT) (4, 9.1%) [19,22,43,49]), and Castration Resistance-Free Survival (CRFS) (1, 2.3%) [47].

A total of 10,631 patients were analyzed across all studies. The sample sizes across studies varied widely, ranging from single-patient mCRPC case reports [20,25,37,38,50] to large-scale clinical trials involving up to 2462 patients with advanced PCa, including metastatic and non-metastatic disease, as well as CRPC and mCSPC [53]. The median age of patients across studies ranged from 39 [25] to 94 years [18], with most studies reporting median ages between 70 and 75 years. However, 17 studies [17,21,30,31,33,43,45,46,47,48,51,52,54,55,56,57,58] did not provide explicit age distribution data.

PSA levels at diagnosis or follow-up varied significantly among studies. Wyatt et al. [29] reported a PSA ranging from 3.4 to 4478 ng/mL, whereas Yuan et al. [37] described a patient whose PSA decreased from 787 ng/mL at diagnosis to 8.02 ng/mL at best response, rising again to 601 ng/mL upon treatment resistance. Similarly, Fettke et al. [60] observed PSA values ranging from 0.51 to 2719 ng/mL, highlighting the heterogeneity of disease burden among study populations.

### 3.4. Methods for Specimen Detected

Sample types, detection methods, and characteristics of genomic alterations and aspect types are summarized in the Supplementary Material (Table S7).

Blood plasma ctDNA was the most primary sample type for testing to identify actionable germline and/or somatic alterations [18,19,20,21,22,23,25,26,27,28,29,30,31,32,33,34,35,36,37,38,39,40,41,42,43,44,45,46,47,48,49,50,52,53,54,55,56,57,58,59,60,61,62]. Analysis of ctDNA in both plasma and urine samples has been performed in only one study [30]. Sample type was not specified in one study [18]. Using plasma rather than serum minimizes the dilution of tumor-derived DNA with leukocyte cfDNA, thereby enhancing detection sensitivity, especially for low variant allele frequency (VAF) mutations [63,64,65,66,67].

Serum samples are more likely to miss alterations with low VAFs, with up to 44.8% of such alterations undetected compared to matched plasma, highlighting the superiority of plasma-based testing [68,69,70,71,72,73,74,75,76,77,78,79,80]. 

**Figure 3:**
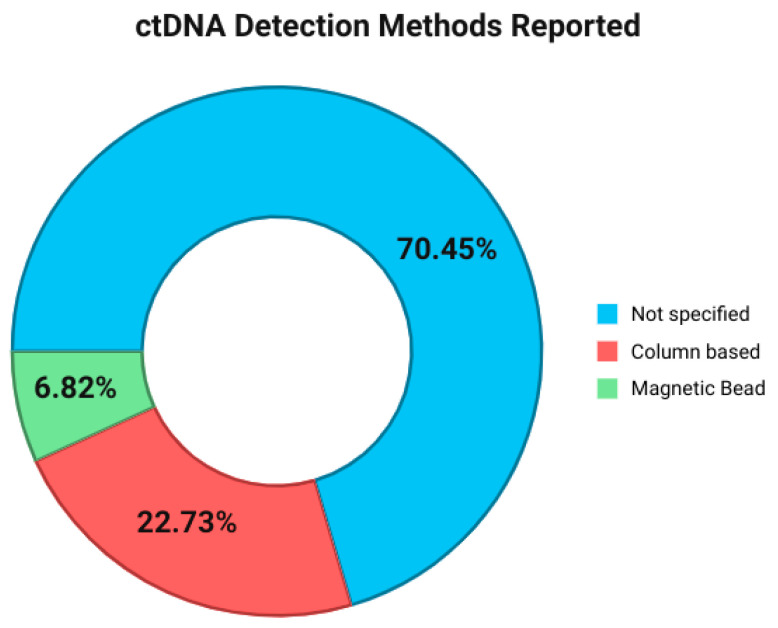
Methods of ctDNA extraction reported in included studies. Donut chart illustrating the methods used for ctDNA extraction among the included studies (n = 44). The majority did not specify the extraction protocol (31/44, 70.5%). Of those reporting a method, most used column-based kits (10/44, 22.7%), while a smaller fraction employed magnetic bead-based extraction (3/44, 6.8%).

For ctDNA extraction, the majority of studies (31, 70.45%) did not explicitly mention the DNA extraction method [18,19,20,22,24,25,26,28,30,32,35,37,38,39,40,41,42,43,46,47,48,49,50,52,53,56,57,58,59,60,61], while the remaining studies (13, 29.55%) reported to have commonly used either column-based or magnetic bead DNA extraction methods [21,23,27,29,31,33,34,36,44,45,51,54,55] (Figure 3). A column-based DNA extraction kit was used in 10 (22.73%) studies [21,23,29,30,34,36,40,44,51,54,59] (Figure 3). The remaining (3, 6.82%) studies used various magnetic bead extraction kits [31,33,45] (Figure 3). Column-based methods typically yield higher cfDNA recovery but capture larger DNA fragments, whereas bead-based methods favor shorter fragments and may provide lower yields [81,82,83,84,85,86,87,88,89,90,91,92]. These differences can influence assay sensitivity, particularly in settings where ctDNA concentration is low [93,94,95,96,97,98].

**Figure 4:**
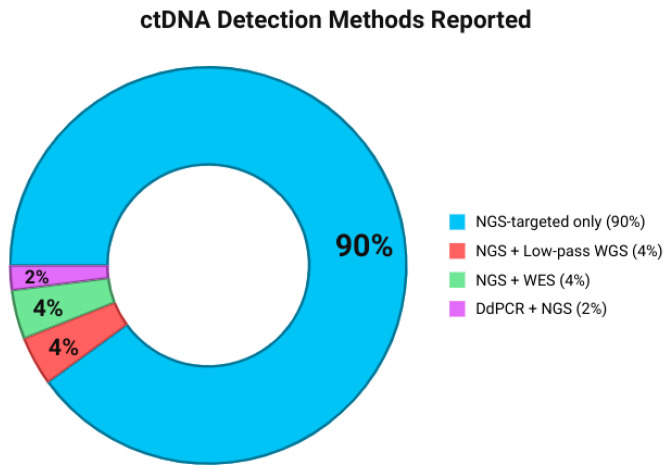
Distribution of ctDNA sequencing methods reported in included studies. Pie chart showing the proportion of ctDNA detection (sequencing) approaches used across the included studies (n = 44). Targeted next-generation sequencing (NGS) alone was the predominant method, while a small number of studies combined NGS with low-pass whole-genome sequencing (WGS) or with whole-exome sequencing (WES). A minority employed digital droplet PCR (ddPCR) in conjunction with NGS.

Nearly every study used next-generation sequencing (NGS) as the primary detection method for ctDNA [18,19,20,21,22,23,25,26,27,28,29,30,31,32,33,34,35,36,37,38,39,40,41,42,43,44,45,46,47,48,49,50,52,53,54,55,56,57,58,59,60,61,62] (Figure 4). Whereas the only study that combined digital droplet PCR (ddPCR) with NGS for targeted ctDNA analysis was performed by Conteduca et al. [41]. All studies that performed NGS used targeted sequencing (40, 91%) [18,19,20,21,22,23,25,26,27,28,29,30,31,32,33,34,35,36,37,38,39,40,41,42,43,44,45,46,47,48,49,50,51,52,53,54,55,59,60], except for two (4.5%) that combined targeted sequencing and low-pass whole-genome sequencing [40,51] and two (4.5%) that combined targeted sequencing and whole-exome sequencing [39,49] (Figure 4).

NGS provides a tumor-agnostic approach that captures the molecular profiles of both primary and metastatic tumors. However, technical challenges remain: low ctDNA concentrations can reduce sensitivity, and accurate detection of low-frequency variants (<0.5–1% VAF) remains difficult [92,99,100,101]. Recent advances such as molecular barcoding and in silico error suppression have improved the reliability of detecting variants with VAFs below 1% [92,101], yet robust performance across all assays is only achieved above ~0.5% VAF. This underscores the importance of using highly sensitive assays and reporting VAF thresholds consistently.

### 3.5. Frequent Genes and Their Somatic/Germline and Genomic Alteration Aspect Type

**Figure 5:**
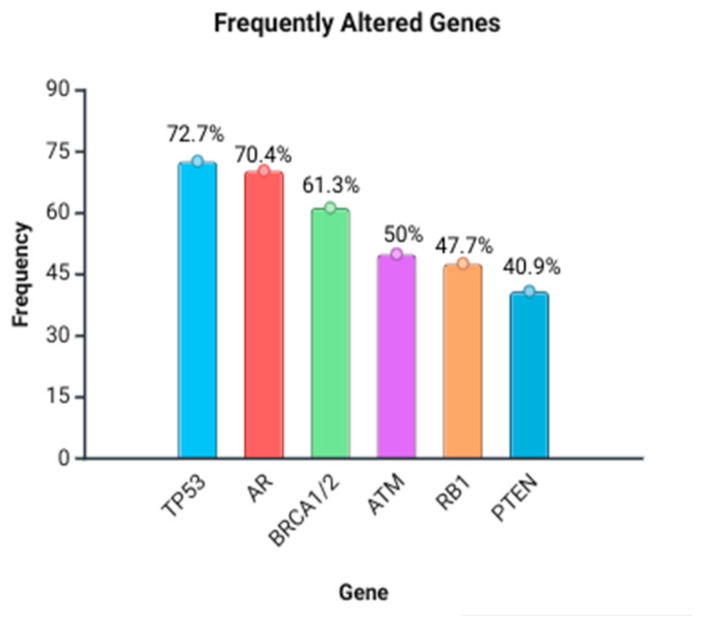
Frequency of gene alterations reported across included studies. *Y*-axis indicates the percentage of studies that reported mutations in a given gene. This does not represent the percentage of patients harboring the mutation.

Blood plasma ctDNA was the most primary sample type for testing to identify actionable germline and/or somatic alterations. Nearly every study used NGS as the primary detection method for ctDNA. TP53 was the most frequently analyzed gene (32, 72.7%) (Table 1, Figure 5) [18,19,21,22,23,25,26,27,28,30,31,32,33,37,38,39,40,44,45,48,49,50,51,53,54,55,56,57,58,61], followed by AR (androgen receptor) (31, 70.4%) [18,20,21,22,23,25,26,27,28,29,30,32,33,34,37,38,39,40,44,45,48,49,51,53,54,55,56,58,59,60,61], BRCA1 and BRCA2 (27, 61.3%) [18,19,21,22,26,27,28,29,31,32,33,34,35,36,38,42,43,44,45,46,47,48,49,52,53,56,57,61], ATM (22, 50%) [18,19,21,22,25,26,27,28,29,30,32,33,34,36,43,44,45,46,48,49,52,53], RB1 (21, 47.7%) [20,21,29,30,32,33,38,39,44,45,48,49,50,51,53,55,60], and PTEN (18, 40.9%) [18,21,22,29,32,33,36,37,39,45,48,51,53,54,55,58,60]. BRCA1, BRCA2, ATM, and CHEK2 were the most common genes (61.4%) that exhibited both somatic and germline alterations. The remaining exhibited either somatic (TP53: 72.7%, AR: 70.5%, and RB1: 47.7%) or germline (PTEN: 40.9%) mutations, with somatic being more prevalent.

Somatic inactivating mutations and deletions in TP53 were observed in nearly all included publications [18,19,20,21,22,23,24,26,27,28,29,30,31,32,33,34,35,36,37,38,39,40,41,42,43,44,45,46,47,48,49,50,52,53,54,55,56,57,58,59,60,61,62]. When specific mutation sites are reported, they often include hotspot changes (e.g., R248Q and R273H) or large deletions spanning multiple exons, all of which disrupt the tumor-suppressor function of TP53 [18,19,20,21,22,23,24,26,27,28,29,30,31,32,33,34,35,36,37,38,39,40,41,42,43,44,45,46,47,48,49,50,52,53,54,55,56,57,58,59,60,61,62]. AR aberrations—spanning somatic amplifications, point mutations (e.g., L702H, T878A, V716M, W742C, and W742L), and structural rearrangements—are some of the most reported [18,20,21,22,23,26,27,28,29,30,31,32,33,34,35,36,37,38,39,40,41,43,44,45,46,47,48,49,52,53,54,55,56,58,59,60,61,62]. In BRCA1/2, germline and somatic alterations were reported, including frameshift mutations, nonsense mutations, and large genomic rearrangements (LGRs) [18,19,20,21,23,24,26,27,28,29,31,32,33,34,35,36,37,38,39,40,41,42,43,44,45,46,47,48,49,50,52,53,54,55,56,57,58,59,60,61,62]. Alterations in ATM encompassed both somatic and germline events, including missense mutations, splice site mutations, and LGRs [18,19,20,21,23,24,26,27,28,29,31,32,33,34,35,36,37,38,39,40,41,43,44,45,46,47,49,50,52,53,54,55,56,57,58,59,60,61,62]. As for RB1, alterations are mainly somatic deletions and inactivating mutations [18,19,20,21,22,23,24,26,27,28,29,30,31,32,33,34,35,36,37,38,39,40,41,42,43,44,45,46,47,48,49,50,52,53,54,55,56,57,58,59,60,61,62]. Somatic deletions, copy number losses, and inactivating point mutations were the commonly observed in PTEN [18,19,20,21,22,23,24,26,27,28,29,30,31,33,34,35,36,37,38,39,40,41,42,43,44,45,46,47,48,49,50,52,53,54,55,56,57,58,59,60,61,62]. Finally, somatic activating point mutations and amplifications were the most frequently observed in the PIK3CA gene [18,19,20,21,22,23,24,26,27,28,29,30,31,32,33,34,35,36,37,38,39,40,41,42,43,44,45,46,47,48,49,50,52,53,54,55,56,57,58,59,60,61,62]. Copy number variations (CNVs) (CNG/amplifications or losses/deletions) are common in genes such as TP53, AR, RB1, PTEN, and MYC [26,28,31,37,39,44,45,48,49,55,59,61]. Frameshift and nonsense mutations have been especially reported in DNA repair genes like BRCA1/2, ATM, CHEK2, PALB2, and FANCA [19,28,32,36,37,38,49,52]. In the case of AR, multiple ligand-binding domain positions are repeatedly mutated, such as L702H, T878A, V716M, and H875Y [26,28,41,44,49,60]. Additionally, gene fusions (e.g., TMPRSS2-ERG) and various LGRs (particularly in BRCA1/2 and ATM) are also reported [23,31,38,43,49,50]. Additional changes and mutations were also reported.

### 3.6. Association of ctDNA Detection with Outcome Survival

**Figure 6:**
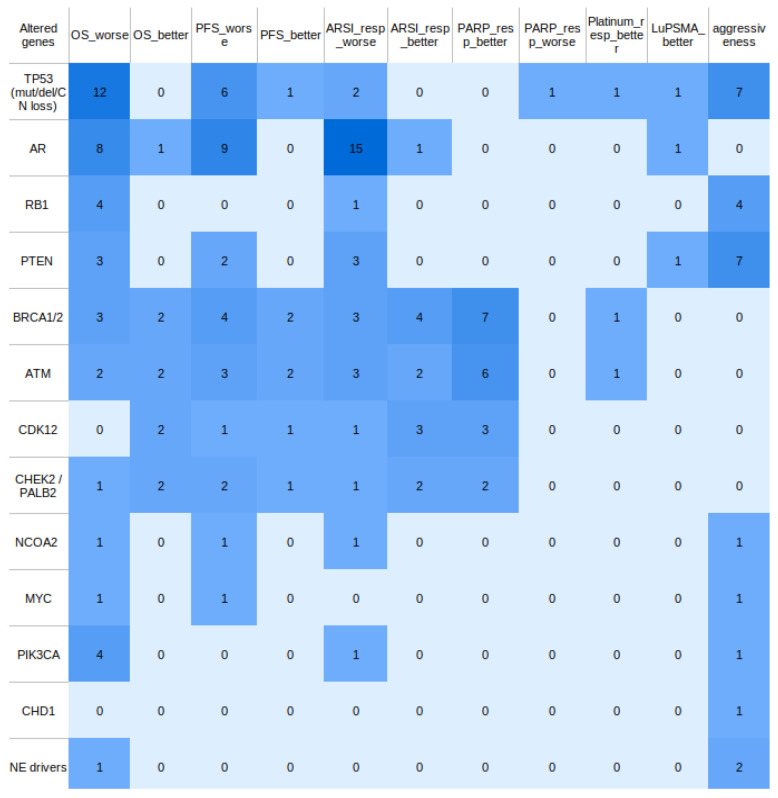
Cross-study heatmap of ctDNA-detected genomic alterations versus clinical endpoints in prostate cancer (44 studies). Heatmap showing study counts linking specific ctDNA-detected alterations (rows: e.g., TP53, AR, RB1, PTEN, BRCA1/2, ATM, CDK12, CHEK2/PALB2, etc.) with clinical endpoints (columns): OS better/worse, PFS better/worse, ARSI response better/worse, PARP response better/worse, platinum response better, 177Lu-PSMA response better, and aggressiveness. Darker shading indicates more studies reporting that association. Counts are study level, not patient level; details in Supplementary Table S8.

The associations between ctDNA-detected genomic alterations and survival outcomes (Table 2) as well as therapy response (Table 3) are presented in simplified form for the main text. A more detailed version of these associations is provided in Supplementary Table S8. A cross-study summary heatmap of gene–endpoint associations is shown in Figure 6.

Impact of ctDNA state on OS: Here, 16 studies [18,21,23,27,39,40,41,44,45,46,47,48,51,55,59,60] totaling 3193 patients reported a relationship between the ctDNA state and OS in PCa patients, including 2917 with mCRPC [18,21,23,27,40,41,44,45,46,47,51,55,60], 250 with mHSPC [44], and 63 with AVPC [39]. Oya et al. [46] investigated the combined therapy of two molecules—Olaparib and Abiraterone—in which they screened patients’ ctDNA to detect Homologous Recombination Repair (HRR) genes (BRCA1/2, ATM, CDK12, PALB2, and RAD51B/D). In this part of the PROpel trial, the combination of Olaparib plus Abiraterone demonstrated a trend toward improved OS in the intention-to-treat population of patients with mCRPC. Specifically, the median OS was 42.1 months for the Olaparib plus Abiraterone group compared to 34.7 months for the placebo plus Abiraterone group, with an HR of 0.81 (95% CI: 0.67–1.00; *p* = 0.0544). Notably, in the subgroup of patients with BRCA1/2 mutations, the survival benefit was more pronounced. In this group, the median OS was not reached in the Olaparib plus Abiraterone arm, whereas it was 23.0 months in the placebo plus Abiraterone arm, corresponding to an HR of 0.29 (95% CI: 0.14–0.56). Not reaching the OS median means that more than half of the patients in that group were still alive at the time of the analysis, implying a strong survival benefit from the combined treatment [46]. Agarwal et al. [48] worked on the combination of Apalutamide + ADT. Genes screened via ctDNA were AR, TP53, PTEN, RB1, PIK3CA, and HRR genes (BRCA2, ATM, etc.). The result was AR amplification at baseline, HR 1.9 or 6.7, all *p* < 0.05) for poor OS [48]. Clarke et al. [47] investigated the efficacy of Olaparib vs. SOC and resulted in OS (not reached) benefit in HRR+ (BRCA1/2: HR 0.29; CI: 0.14–0.56) upon screening BRCA1/2, ATM, CDK12, and CHEK2 (HRR pathway) genes in association with OS [47]. Knutson et al. [55] investigated ARPI combinations via screening AR, TP53, RB1, PTEN, MYC, and MYCN genes, resulting in worse OS for AR alterations (e.g., LBD truncations) [55]. De Bono et al. [58] evaluated 177Lu-PSMA-617 vs. ARPI and reported that AR, TP53, and PTEN alterations detected in patients’ ctDNA were associated with shorter rPFS (AR: HR 1.954, 95% CI 1.333–2.865, and *p* < 0.001; TP53: HR 1.655, 95% CI 1.13–2.426, and *p* < 0.01; PTEN: HR 1.62, 95% CI 1.018–2.578, and *p* < 0.05) [58]. Jayaram et al. [45] explored the combined therapy of Apalutamide + ADT and reported worse OS (TP53: HR 7.13, 95% CI 2.37–21.47, and *p* < 0.001; RB1: HR 6.24, 95% CI 1.97–19.73, and *p* = 0.002; PTEN: HR 11.9, 95% CI 3.6–39.34, and *p* < 0.001) when persistent TP53/RB1/PTEN alterations were screened in ctDNA [45].

Impact of ctDNA state on PFS: Here, 21 studies [21,23,34,35,36,37,39,40,41,45,46,47,48,49,52,54,55,57,58,59,60] involving 3783 patients reported on the association between the ctDNA state and PFS, including 3099 with mCRPC [21,23,36,40,45,46,47,49,52,54,55,57,58], 265 with CRPC [41], 161 with nmPC [35], 129 with mCSPC [48], and 63 with AVPC [39]. In the PROpel NCT03732820 study [47], Abiraterone combined with Olaparib significantly prolonged PFS compared with Abiraterone and placebo as first-line treatment for patients with mCRPC enrolled irrespective of HRRm status (HR 0.50, 95% CI: 0.34–0.73), with the greatest benefit in BRCA1/2-mutated subgroups (HR 0.23, 95% CI: 0.12–0.43). In the TITAN study [43], Apalutamide plus ADT improved OS, delayed castration resistance, maintained health-related quality of life, and improved radiographic PFS in a broad population of patients with mCSPC compared to the control. The PROpel study [56], which focused on patients with mCRPC, showed that Abiraterone combined with Olaparib significantly improved rPFS compared to Abiraterone plus placebo (HR 0.66, 95% CI: 0.54–0.81, *p* < 0.001). In the Alliance A031201 study [55], which analyzed ARPI combinations in mCRPC, AR alterations predicted worse rPFS, emphasizing their role in resistance. In the TITAN ctDNA study [45], the detection of ctDNA pre-treatment was significantly associated with shorter PFS (HR: 2.05, 95% CI: 1.36–3.11, and *p* < 0.0002). Multivariable analysis confirmed that ctDNA positivity remained an independent prognostic factor for PFS after adjusting for established clinical variables (HR 2.03, 95% CI: 1.21–3.41, and *p* < 0.007). The PSMAfore NCT04689828 study reported shorter rPFS with AR (HR 1.954; 95% CI 1.333–2.865; *p* < 0.001), TP53 (1.655; 1.13–2.426; *p* < 0.01) and PTEN (1.62; 1.018–2.578; *p* < 0.05) alterations, linking ctDNA profiles to treatment resistance [58]. The ProBio investigation found that TP53-altered patients had inferior PFS (STR 0.76), suggesting limited efficacy of platinum therapy in genomically aggressive disease [57]. An association was observed between a positive ctDNA state and poor PFS in mCRPC followed by mCSPC and AVPC, which highly probably indicates an association between positive ctDNA state and poor PFS in patients with mCRPC [21,23,34,35,36,37,39,40,41,45,46,47,48,49,52,54,55,57,58,59,60].

### 3.7. Discussion

Understanding genomic landscapes of PCa is important, owing to the emergence of PM to guide treatment selection and improve survival outcomes. Integrated genomic and proteogenomic characterization of prostate tumors identifies biological insights and subtype-specific therapeutic strategies. ctDNA profiling provides non-invasive access to a malignancy’s molecular landscape, overcoming the limitations of tissue biopsy [64,76]. In cases where archival tissue is not available for testing and tissue-based analysis is not clinically feasible, NGS-based HRR gene testing of ctDNA from plasma provides a minimally invasive alternative [29,65,66,67]. There are also inherent advantages in profiling the latest available sample from a patient with advanced disease. Park et al. [68] showed that 11% of mCRPC patients harbored actionable genetic alterations by serial NGS based-ctDNA testing, with 3.5% of tests detecting a new BRCA2 genetic alteration or MSI-high [68]. ctDNA harbored some BRCA1/2 alterations not identified by tissue testing, and it was enriched in therapy resistance alterations, as well as possible clonal hematopoiesis mutations (e.g., in ATM and CHEK2) [78]. mPCa presents challenges in the collection of a tissue specimen since metastases are often confined to bone, requiring a technically difficult, invasive biopsy [71,79]. ctDNA testing holds promise for contributing to improved management of PCa from localized disease to metastatic stages, though its role is not yet fully established in routine practice. Thus, certain technical and biological challenges remain to be addressed before widespread clinical implementation in the field. False negative results can occur due to low levels of ctDNA in the patient’s blood [65]. Disease burden, including a PSA > 10 ng/mL, lymph node-only disease, and normal LDH are strongly associated with detecting somatic mutations in cfDNA NGS studies [72,73]. Clonal hematopoiesis of indeterminate potential (CHIP) can also interfere with ctDNA testing, potentially leading to false positive results. In addition, accurate determination of copy number variations from ctDNA has been shown to be difficult, especially in low-tumor-fraction settings [65]. Because of these challenges, it is important to make ctDNA tests more precise. To move toward clinical integration, clear best practices, standard protocols, and quality measures for ctDNA analysis using NGS are still required. It is also important to collect ctDNA samples at the right time, especially when the cancer is clearly progressing [66,74,75].

Plasma-based ctDNA analysis has shown advantages over serum. Using plasma minimizes dilution of tumor-derived DNA compared to serum, enhancing sensitivity [76]. In serum, leukocyte cfDNA released during clotting dilutes ctDNA, reducing variant allele frequency (VAF) detection [77,78,79]. Up to 44.8% of low-VAF alterations were missed in serum compared to plasma, and concordance with somatic mutations in tissue biopsy was lower [80]. Thus, retrospective studies using serum must consider these limitations. VAFs in ctDNA are being considered one of the markers with prospective clinical utility, and the use of VAFs analysis may provide information about response to treatment and patient prognosis and help in developing optimal therapy. Recent studies demonstrate an association between high VAF level in ctDNA and shorter OS among patients with metastatic disease [81,82,83,84].

In cases where detectable alterations are found in ctDNA, the concordance level between genomic analysis of tumor tissue DNA and blood-derived ctDNA when acquired at the same time can be as high as 80–90% [85]. Shiota et al. [86] showed that the sensitivity of ctDNA testing represented by the ratio of detected gene alterations in blood among those in tissues from mPCa patients was 41.5% [86]. The sensitivities of ctDNA testing in mCSPC and mCRPC were 43.8% and 35.9%, respectively. The concordance status of alterations in the genes with a frequency of over 5% in tissue or ctDNA at pre-treatment among matched patients [86]. Among them, 33.7% alterations were concordant between tissue and ctDNA, whereas 66.3% alterations were discordant. AR alterations were detected only in ctDNA from patients with mCRPC, whereas there was no AR alteration in tissue, which was mostly obtained before hormonal therapy [87]. Simultaneous sequencing of ctDNA and tumor DNA in individuals with metastatic disease results in high levels of concordance between ctDNA and tissue profiling for driver gene analysis, suggesting the potential utility of ctDNA analysis in advanced stages of disease [87]. Wyatt et al. [29] conducted a study on the concordance of ctDNA with time-matched metastatic tissue biopsy in PCa and showed that 33% of somatic mutations were detected exclusively in liquid biopsy samples [29]. In mPC, ctDNA is a high-fidelity substitute for solid tumor tissue-derived DNA and is capable of not only recapitulating the somatic landscape of a tumor but also identifying clinically relevant driver alterations missed by a single metastatic biopsy [29,51,88]. Although archival tissue from primary tumors, including formalin-fixed, paraffin-embedded diagnostic biopsies, would be best for molecular characterization [89,90], this does not usually reflect metastatic disease that has evolved under treatment selective pressure and does not account for intra-patient heterogeneity. For example, metastatic samples are generally enriched for RB1 and TP53 mutations, while treatment-naive PCa tissues are not, but they can harbor HRR defects [91].

The choice of cfDNA extraction method is important in ctDNA studies given reported disparities in cfDNA yield and composition across commonly used kits. In our systematic review, column-based DNA extraction kits were used in 10 (22.73%) studies [21,23,29,30,34,36,40,44,51,54,59]. The remaining studies did not explicitly mention the ctDNA extraction method [18,19,20,22,24,25,26,28,30,32,35,37,38,39,40,41,42,43,46,47,48,49,50,52,53,56,57,58,59,60,61], or used various magnetic bead extraction kits [31,33,45], (31, 70.45%, and 3, 6.82%, respectively). Multiple studies have evaluated the performance of the various cfDNA methods [92,93,94,95,96]. The main methods for cfDNA extraction are the spin column-based method (e.g., QIAamp Circulating Nucleic Acid Kit [Qiagen, Hilden, Germany], Quick-cfDNA Serum & Plasma Kit [Zymo Research, Irvine, CA, USA], and Plasma/Serum Cell-free Circulating DNA Purification Midi Kit [Norgen Biotek Corp., Thorold, ON, Canada]) and the magnetic bead-based method (e.g., QIAamp minElute ccfDNA Mini Kit [Qiagen], Maxwell RSC ccfDNA Plasma Kit [Promega, Madison, WI, USA], MagMAX cell-free DNA Isolation Kit [Thermo Fisher Scientific, Waltham, MA, USA], NextPrep-Mag cfDNA Isolation Kit [Bioo Scientific, Austin, TX, SA], and Magnetic Serum/Plasma Circulating DNA Kit [Dxome, Seoul, Korea]) [97]. Although spin column-based methods are more time consuming and more costly than magnetic bead-based approaches, they typically produce higher yields. Column-based methods showed superior cfDNA yield, albeit with a tendency to capture larger DNA fragments. Magnetic bead-based methods yielded less cfDNA but exhibited a bias toward recovering shorter DNA fragments [98]. There was a high level of consensus among studies regarding both cfDNA extraction methods, with a majority of studies using column-based methods. Several comparative studies report that bead-based kits yield higher DNA recovery rates than column-based kits, particularly at low DNA input, though absolute recovery percentages vary depending on the platform.

In regard to the sequencing method, NGS was the most utilized method accounting for the totality of studies [18,19,20,21,22,23,25,26,27,28,29,30,31,32,33,34,35,36,37,38,39,40,41,42,43,44,45,46,47,48,49,50,52,53,54,55,56,57,58,59,60,61,62]. These assays include amplicon based in 13.63% of studies [21,23,31,36,41,54] and hybridization capture-targeted NGS in 70.45% of studies [19,22,24,25,26,27,28,30,31,32,33,34,37,38,39,42,43,44,45,46,47,49,50,51,52,53,56,59]. The remaining seven (15.9%) studies [35,40,48,57,58,60,61] did not specify the type of NGS used. Both strategies are followed by highly redundant (“deep”) sequencing to allow for the relative amount of mutant and wild-type DNA molecules at each locus to be accurately counted.

NGS offers distinct advantages by allowing a tumor-agnostic approach for cfDNA analysis. This approach permits the identification of variants without the need for molecular profiling of tumor tissue, offering a systemic view of the disease by capturing the molecular profiles of both primary and metastatic tumors. However, one of the most difficult parts of accurately reporting liquid biopsy NGS results is indeed related to ctDNA samples with variants detected at low VAF. For example, Williams et al. [99] showed decreased detection of SNVs at low VAFs when using two allele-specific ddPCR and three (amplicon and hybrid capture) NGS panels [99]. As the ctDNA concentration in the blood is low, most platforms use methods to increase the signal of certain regions to increase the sensitivity of ctDNA detection at low tumoral VAFs [100]. Reliable detection of mutations below 0.5% VAF remains a key challenge for ctDNA tumor DNA sequencing approaches. Recent advancements in NGS technologies have enhanced sensitivity by implementing strategies such as molecular barcoding and in silico error suppression. These innovations allow the reliable differentiation of real mutations with VAFs < 1% from background artifacts [92]. The analytical performance evaluation of NGS-based ctDNA assays has undergone in earlier studies. Deveson et al. [101] evaluated the ctDNA detection performance of five leading ctDNA assay platforms developed based on NGS across 12 clinical and research facilities. Above 0.5% VAF, ctDNA mutations were detected with high sensitivity, precision, and reproducibility by all five assays, whereas below this limit of 0.5%, detection became unreliable, making it a key challenge for ctDNA sequencing analysis [101]. Detecting low-frequency ctDNA variants with a VAF < 1% in CRPC is important to identify early, subclonal, and clinically significant genomic alterations that may not be detectable by traditional methods, thereby improving treatment selection and prognostic risk stratification [102].

In our systematic review, TP53 was commonly detected, followed by alterations in AR, BRCA1/2, ATM, RB1, and PTEN genes. Alterations in TP53 in localized or metastatic hormone-sensitive PCa had a shorter time to CRPC, and cumulative gene hits in TP53, PTEN, and RB1 led to an incremental risk of progression with inferior OS with increasing gene hits [103]. Co-occurrence of alterations in TP53 and other important tumor suppressors, in particular RB1 or PTEN, was shown to render PCa tumor cells more aggressive or more resistant towards conventional therapies. In neuroendocrine PCa, the concomitant alteration of TP53 and RB1 is linked to aggressive clinical features [104] and responsiveness to platinum treatments [105]; however, it could be present also in CSPC, where is associated with increased risk of relapse and death [105]. In AR, W742C, W742L, L702H, and T878A were the most frequent points mutations detected. These hotspot mutations were revealed to change the binding affinity of ligands, including steroids and antiandrogens, and potentially result in altered responses to AR pathway inhibitors. W742C and W742L are associated with resistance to Bicalutamide/Enzalutamide by causing the AR antagonists to behave as agonists [106]. L702H is associated with resistance to Abiraterone/Enzalutamide and promotes the trans-activation of AR by glucocorticoids [89]. T878A is associated with resistance to Bicalutamide/Enzalutamide/Apalutamide and promiscuous activation by progesterone. The frequency of alterations in AR and HRR genes, including BRCA2 and ATM, was enriched in mCRPC compared with mCSPC in the most studies included in this review. These finding suggested that alterations in AR and HRR genes promote treatment resistance to initial ADT-based therapy in PCa [86]. Fan et al. [32] have shown that genomic alterations in AR and CDK12 genes were enriched in ctDNA with mCRPC compared with those with de novo mCSPC among Asians [32]. Previous studies showed that genomic alterations in AR, TP53, RB1, and PTEN are enriched during PCa progression [90,107,108]. Shaya et al. [18] found that mCRPC patients with more than one alteration on ctDNA analysis have inferior OS (26.1 months vs. 8.8 months, *p* < 0.001), suggesting that the presence and actionable alterations identified by NGS can predict OS in mCRPC [18].

Our analysis highlights the prognostic and predictive value of ctDNA in localized and metastatic PCa, particularly in the mCRPC setting. Across 16 studies evaluating OS and 21 studies examining PFS, a consistent trend emerged: ctDNA positivity or the presence of specific genomic alterations detected via ctDNA was significantly associated with worse clinical outcomes. The consistent association between positive ctDNA states and poor OS and PFS supports ctDNA as a non-invasive biomarker of disease aggressiveness. For example, alterations in TP53, RB1, and PTEN—frequently associated with poor prognosis—were strongly linked with significantly reduced survival (e.g., TP53: HR 7.13; PTEN: HR 11.9) [45], underscoring the utility of ctDNA profiling in risk stratification. Additionally, AR amplifications and truncations were associated with inferior OS and rPFS, indicating resistance to AR pathway inhibitors (ARPI) [48,55]. Other studies within our review, such as PROpel and TITAN, demonstrated that ctDNA profiling can inform therapeutic decision making. In PROpel, patients with BRCA1/2 mutations benefited significantly from Olaparib + Abiraterone combination therapy (HR for OS: 0.29) [46,47], illustrating how HRR mutations identified via ctDNA can guide the use of PARP inhibitors. Patients with BRCA1/2 mutations, who are often sensitive to therapies like PARP inhibitors, can develop acquired resistance when their cancer acquires new reversion mutations. These reversion mutations can restore a functional BRCA1/2 gene, allowing the cancer cells to repair DNA damage and continue growing, thus nullifying the effect of the combination therapy and leading to treatment resistance. This emergence of new mutations is a significant mechanism of secondary resistance in these cancers. Conversely, patients with AR, TP53, and PTEN alterations experienced limited benefits from standard therapies, suggesting a need for alternative regimens in these subgroups [55,58]. The association of AR, TP53, and PTEN alterations with shorter rPFS in several trials reflects emerging patterns of treatment resistance. These findings imply that ctDNA could serve not only as a baseline predictor but also as a real-time monitor of evolving resistance, enabling dynamic treatment adaptation. For example, the observation of persistently altered ctDNA profiles pre- and post-treatment in the TITAN study correlated with poor PFS, suggesting potential for ctDNA to inform early treatment modification [45]. The results of clinical implications and integration suggest that ctDNA-based genomic profiling has the potential to upgrade clinical management of metastatic PCa, with further evidence required for its full adoption into routine practice. Its use could (i) identify patients likely to benefit from targeted therapies (e.g., BRCA-mutated patients for PARPi), (ii) detect high-risk molecular subtypes (e.g., TP53/PTEN/RB1 alterations), and (iii) monitor treatment response and resistance emergence over time. Moreover, ctDNA offers an attractive alternative to invasive tissue biopsies, especially when tumor accessibility is limited, and may facilitate more frequent and patient-friendly molecular assessments. Importantly, this work was conducted by a Moroccan team as part of a national effort to lay the groundwork for the integration of ctDNA technologies in PCa diagnostics and treatment pathways. As a first of its kind in the region, this review aspires not only to inform clinical practice but also to ignite a larger research and policy momentum that may accelerate the adoption of PCa precision oncology in Morocco and the broader African context. In a field largely driven by data from high-income countries, this review highlights the potential for scientific leadership to emerge from North Africa, guided by both local needs and global standards.

### 3.8. Practical Recommendations

We have added a new “Practical Recommendations” subsection summarizing translational insights and clinical take-home points.

Based on the synthesis of 44 studies (10,631 patients) included in this review, we propose several practice-oriented recommendations for clinicians and researchers working with ctDNA in prostate cancer:

- Sample type and handling: Plasma should be used rather than serum to minimize dilution by leukocyte cfDNA and to improve detection sensitivity. Pre-analytical variables (time to centrifugation, storage temperature, and number of freeze–thaw cycles) must be standardized to avoid degradation.
- DNA extraction: Bead-based kits generally yield higher recovery rates than column-based methods, particularly when ctDNA concentration is low. We recommend reporting recovery efficiency in each study to facilitate comparability.
- Sequencing approaches: Next-generation sequencing (NGS) with unique molecular identifiers (UMIs) and error-suppression algorithms should be preferred for detecting low-frequency variants (<1% VAF). Targeted panels focusing on recurrent alterations (TP53, BRCA2, AR, and PTEN) are currently the most practical for clinical monitoring.
- Interpretation of results: Variants associated with clonal hematopoiesis (e.g., ATM, CHEK2, and DNMT3A) should be carefully interpreted in parallel with matched white blood cell sequencing to reduce false positives.
- Clinical integration: At present, ctDNA analysis is most useful to (i) identify resistance mechanisms to androgen receptor signaling inhibitors (ARSI), (ii) guide the addition of PARP inhibitors in patients with Homologous Recombination Repair (HRR) alterations, and (iii) monitor emerging mutations during treatment.
- Reporting standards: Studies should systematically report the variant allele fraction (VAF), copy number thresholds, and assay sensitivity. Uniform reporting will accelerate meta-analyses and guideline development.

### 3.9. Limitations

This study has some limitations. Although 44 articles were included in our systematic review, a number of relevant studies had to be excluded due to insufficient data. Many lacked critical information, such as the clinical significance of specific genomic alterations, hazard ratios, and confidence intervals, which prevented us from conducting a comprehensive meta-analysis. Additionally, in several studies, ctDNA sequencing was performed on only a subset of enrolled patients, rather than the entire cohort. Some patients had only tissue biopsies without corresponding liquid biopsy data, limiting the ability to assess concordance between tissue and ctDNA findings and potentially biasing conclusions regarding their equivalence. We also came across studies that explored associations between ctDNA levels or fractions and treatment resistance or response; however, these were outside the scope of our research question. Furthermore, none of the included studies enrolled participants of African, Middle Eastern, or other under-represented ethnic backgrounds. This lack of diversity is a notable limitation, as these populations remain significantly under-represented in prostate cancer research despite their relevance. Limited to no representation from African and Middle Eastern populations is considered a critical gap that must be addressed in future research to ensure global applicability of ctDNA-based strategies.

## 4. Conclusions and Clinical Implications

In this systematic review, we evaluated the relationship between genetic alterations detected through ctDNA sequencing and clinical outcomes in localized and metastatic prostate cancer.

Prognostic value: ctDNA positivity and alterations in TP53, AR, BRCA1/2, PTEN, and RB1 consistently correlate with poorer OS, shorter PFS, and resistance to therapy, particularly in mCRPC. Predictive value: HRR alterations (e.g., BRCA1/2) can guide treatment with PARP inhibitors, while AR pathway alterations predict resistance to AR-targeted therapies. Clinical utility: ctDNA profiling thus provides clinically meaningful information to (a) stratify prognosis, (b) guide personalized therapy selection, and (c) monitor disease progression over time. These findings directly address the main objective of our study and support the promising role of ctDNA—which requires further consolidation through standardized validation and broader clinical evidence—in advancing prostate cancer management as a non-invasive tool for precision oncology.

A significant limitation in the clinical application of ctDNA lies in the absence of unified guidelines from leading oncology societies such as National Comprehensive Cancer Network (NCCN), The American Society of Clinical Oncology (ASCO), and The European Society for Medical Oncology (ESMO). Currently, there is no standardized protocol for PCa ctDNA sample processing, sequencing methods, or result interpretation, which leads to variability across studies and challenges in cross-comparison. Furthermore, ctDNA assays exhibit technical limitations in detecting certain genomic alterations in PCa, particularly long-range reads (LRGs) and copy number variations. The LRGs often pose detection challenges due to fragmentation and low abundance in plasma, potentially resulting in missed clinically relevant targets. Addressing these gaps requires the development of harmonized workflows and improvements in assay sensitivity to fully harness the potential of ctDNA as a comprehensive biomarker in PCa.

Precision medicine is an approach that tailors treatment and prevention strategies to the individual, based on their genetic, molecular, environmental, and lifestyle characteristics, but this only works if we understand the molecular landscape across all populations. Currently, approximately 18–19% of the world’s population lives in Africa, and the Middle East and North Africa (MENA) region has around 500 million people, or about 6–7% of global population. Yet in our review, no study included substantial representation from African or Middle Eastern cohorts. This is not a minor gap: genetic variation differs substantially among populations, and findings drawn mainly from European, North American, or East Asian groups may not generalize to regions with different ancestries. For example, allele frequencies, variant pathogenicity, linkage disequilibrium, and response to therapies (both in efficacy and adverse effects) can vary by population. Under--representation of African and Arab populations means that an entire ~25–30% of humanity is largely invisible to the genomic studies upon which precision oncology depends. Without more inclusive research—whole-genome sequencing, variant databases, local biobank efforts in Africa and the Middle East—we risk treating only a subset of patients well while leaving others behind. Expanding diversity is essential not just for equity, but for unlocking novel variants, improving prediction models, and increasing therapy success rates globally.

Despite promising findings, several challenges remain: heterogeneity in PCa ctDNA assay platforms and panels (e.g., 72-gene panels vs. broad 585-gene assays) limits cross-study comparability. The clinical utility of ctDNA in PCa needs validation in prospective, randomized studies. Standardization of ctDNA thresholds and reporting is essential to integrate it into guidelines regarding PCa patient care.

## Figures and Tables

**Table 1:** Spectrum of genomic alterations in prostate cancer: frequency and alteration types across included studies.

Gene	Frequency of Reports (n Studies)	Common Alteration Types Reported
TP53	32/44	Missense, deletions, loss of function, and copy number loss
AR	31/44	Amplifications (CNG), ligand-binding domain mutations (L702H, T878A, H875Y, W742C/L, and F877L), and rearrangements
BRCA1/2	27/44	Germline and somatic truncating, frameshift, deletions, reversion mutations, pathogenic variants, and nonsense
ATM	22/44	Germline and somatic missense, truncations, and deletions
PTEN	18/44	Copy number loss, deletions, and inactivation
RB1	21/44	Deletions, mutations, and rearrangements
CDK12	9/44	Mutations and biallelic loss
PIK3CA	13/44	Missense mutations and amplifications
MSI-H/MMR genes (MSH2, MSH6, MLH1, and PMS2)	6/44	Frameshift, loss, and microsatellite instability
SPOP	3/44	Point mutations
MYC	6/44	Amplifications and mutations
APC	7/44	Mutations
Others (e.g., PALB2, CHEK2, FANCA, NCOR2, FOXA1, BRAF, EGFR, MET, FGFR1/2/3/4, ERBB2, IDH1, HOXB13, etc.)	≤3/44 each	Various rare mutations or copy number changes

CNG, Copy Number Gain.

**Table 2:** Alterations associated with survival (OS/PFS).

Gene/Pathway	Alteration Type	Clinical Association (OS/PFS)	References
TP53	Mutations, deletions, and copy number loss; specific variants (e.g., c.665_672*11del)	Worse OS and/or PFS; some studies report platinum sensitivity despite poor prognosis	[18] (trend), [14,21,23,37,39,40,44,45,48,51,55,57]
PTEN	Loss/deletion and inactivation; frameshift (e.g., p.Y46Qfs*5)	Shorter OS/poor prognosis; aggressive phenotype	[39,45,48,51,60]
RB1	Loss/deletion and mutations	Shorter OS; adverse prognosis; lineage plasticity features	[39,44,45,51,55]
AR (amplifications/CNV, SNVs, and GSRs)	CN gain; LBD SNVs (L702H, T878A, H875Y, W742C/L, and F877L); structural rearrangements	Shorter OS and/or rPFS in multiple studies; early progression on ARSIs in some cohorts	[14,18,37,40,41,44,55,59]
PIK3CA/PI3K pathway	Mutations and CN gain/amplification	Worse OS/PFS; aggressive disease biology	[21,45,48]
HRR genes (BRCA1/2, ATM, CDK12, CHEK2, PALB2, etc.)	Deleterious/truncating/germline and somatic	Worse OS/PFS on ARSI; prognostic effect heterogeneous across genes	[14,36,44] (worse PFS on Abiraterone HRRmt); (therapy benefit details placed in Table S8 Supplementary Material)
NCOA2	Copy number gain; missense	Significantly shorter OS and PFS	[60]
MYC	Copy number gain/amplification	Poorer outcomes in some datasets; neutral in others	[55] (poorer); [44] (no clear link); [51] (N/A survival stated)
MYCN	Copy number gain	Associated with adverse outcomes/AVPC features	[55]
TMPRSS2-ERG	Fusion	No explicit OS/PFS link	[23]
CHD1	Loss/deletion	Worse metastasis-free survival noted contextually; OS/PFS not clearly quantified	[37]

**Table 3:** Alterations associated with therapy resistance/response.

Gene/Pathway	Alteration Type	Therapy Association	References
AR	CN gain/amplification; LBD SNVs (L702H, T878A, F877L, H875Y, and W742C/L); GSRs; enhancer amplification	Resistance to ARSIs (Enzalutamide/Abiraterone); shorter response duration; primary resistance with AR-GSRs; enhancer/gene body amp linked to poor ARSI outcomes	[14,18,21,23,40,41,59]
TP53 + RB1 (±PTEN) co-alteration	Co-loss/combined alterations	Lineage plasticity/neuroendocrine-like features; ARSI resistance; highly aggressive biology	[21,39,51,55]
PTEN	Deletion/loss	Poor response to AR-targeted therapy; aggressive course	[51,58,60] (poor ARPI rPFS)
HRR genes (BRCA1/2, ATM, etc.)	Pathogenic/truncating (germline and somatic)	PARP inhibitor benefit; greatest with BRCA1/2; mixed/limited benefit with non-BRCA HRR	[14,46,47,52] (poor ARSI outcomes)
PALB2	Pathogenic + reversion mutations	Initial PARPi sensitivity; reversion mutations → acquired PARPi resistance	[37]
PMS2/MMR	Pathogenic mutation/MSI-H	May benefit from checkpoint inhibitors (e.g., pembrolizumab)	[18,22]
CDK12	Mutations/biallelic loss	High TMB → checkpoint inhibitor sensitivity; limited PARPi benefit	[34,52]
PI3K pathway (PIK3CA and PTEN loss context)	Mutations/CN gain	AR-targeted therapy resistance; rationale for PI3K/AKT/mTOR combinations	[14,21,48]
Therapy-modality signal (Lu-PSMA)	AR, TP53, and PTEN alterations	Poorer rPFS on ARPIs but better rPFS with 177Lu-PSMA-617 vs. changing ARPI	[58]
Platinum sensitivity signal	TP53 alterations	Poor OS overall but better response to platinum chemo in AVPC context	[39]
NCOA2	CN gain/missense	Poor ARPI outcomes (no PSA Responses when gain present)	[60]

## Data Availability

All data generated or analyzed during this study are included in this published article.

## Supplementary Materials

The following supporting information can be downloaded at: https://www.mdpi.com/article/10.3390/ijms262211049/s1.
