# Supplementary material for: Circulating Tumor DNA as a Biomarker for Precision Medicine in Prostate Cancer: A Systematic Review
Source: Int J Mol Sci. 2025 Nov 15;26(22):11049. doi: 10.3390/ijms262211049 (PMC12652532; doi:10.3390/ijms262211049)
Supplement: Supplementary file 1 [file ijms-26-11049-s001.zip › In_Manuscript_Table_1.pdf]

Table 1: Spectrum of genomic alterations in prostate cancer: frequency and alteration types across included studies.

Gene	Frequency of reports (n studies)	Common alteration types reported
TP53	32/44	Missense, deletions, loss-of-function, copy number loss
AR	31/44	Amplifications (CNG), ligand-binding domain mutations (L702H, T878A, H875Y, W742C/L, F877L), rearrangements
BRCA1/2	27/44	Germline & somatic truncating, frameshift, deletions, reversion mutations, pathogenic variants, nonsense.
ATM	22/44	Germline & somatic missense, truncations, deletions
PTEN	18/44	Copy number loss, deletions, inactivation
RB1	21/44	Deletions, mutations, rearrangements
CDK12	9/44	Mutations, biallelic loss
PIK3CA	13/44	Missense mutations, amplifications
MSI-H / MMR genes (MSH2, MSH6, MLH1, PMS2)	6/44	Frameshift, loss, microsatellite instability
SPOP	3/44	Point mutations
MYC	6/44	Amplifications, mutations
APC	7/44	Mutations
Others (e.g., PALB2, CHEK2, FANCA, NCOR2, FOXA1, BRAF, EGFR, MET, FGFR1/2/3/4, ERBB2, IDH1, HOXB13, etc.)	≤3/44 each	Various rare mutations or copy number changes

CNG, Copy Number Gain.
