# Supplementary material for: Circulating Tumor DNA as a Biomarker for Precision Medicine in Prostate Cancer: A Systematic Review
Source: Int J Mol Sci. 2025 Nov 15;26(22):11049. doi: 10.3390/ijms262211049 (PMC12652532; doi:10.3390/ijms262211049)
Supplement: Supplementary file 1 [file ijms-26-11049-s001.zip › In_Manuscript_Table_2 and_Table_3.pdf]

Table 2: Alterations associated with Survival (OS / PFS)

Gene / pathway	Alteration type	Clinical association (OS/PFS)	References
TP53	Mutations, deletions, copy-number loss; specific variants (e.g., c.665_672*11del)	Worse OS and/or PFS; some studies report platinum sensitivity despite poor prognosis	[18] (trend), [14], [30], [23], [44], [45], [51], [48], [41], [55], [57], [39], [21]
PTEN	Loss/deletion, inactivation; frameshift (e.g., p.Y46Qfs*5)	Shorter OS / poor prognosis; aggressive phenotype	[51], [60], [45], [48], [41]
RB1	Loss/deletion, mutations	Shorter OS; adverse prognosis; lineage plasticity features	[44], [45], [51], [41], [55]
AR (amplifications/CNV, SNVs, GSRs)	CN gain; LBD SNVs (L702H, T878A, H875Y, W742C/L, F877L); structural rearrangements	Shorter OS and/or rPFS in multiple studies; early progression on ARSIs in some cohorts	[18], [31], [14], [59], [30], [44], [55], [39]
PIK3CA / PI3K pathway	Mutations, CN gain/amplification	Worse OS/PFS; aggressive disease biology	[21], [45], [48]
HRR genes (BRCA1/2, ATM, CDK12, CHEK2, PALB2, etc.)	Deleterious/truncating/germline & somatic	Worse OS/PFS on ARSI; prognostic effect heterogeneous across genes	[14], [44], [38] (worse PFS on abiraterone HRRmt); (therapy-benefit details placed in Table 8 supplementary material)
NCOA2	Copy-number gain; missense	Significantly shorter OS and PFS	[60]
MYC	Copy-number gain/amplification	Poorer outcomes in some datasets; neutral in others	[55] (poorer); [44] (no clear link); [51] (N/A survival stated)
MYCN	Copy-number gain	Associated with adverse outcomes / AVPC features	[55]
TMPRSS2-ERG	Fusion	No explicit OS/PFS link	[23]
CHD1	Loss/deletion	Worse metastasis-free survival noted contextually; OS/PFS not clearly quantified	[39]

Table 3: Alterations associated with Therapy Resistance / Response

Gene / pathway	Alteration type	Therapy association	References
AR	CN gain/amplification; LBD SNVs (L702H, T878A, F877L, H875Y, W742C/L); GSRs; enhancer amplification	Resistance to ARSIs (enzalutamide/abiraterone); shorter response duration; primary resistance with AR-GSRs; enhancer/gene-body amp linked to poor ARSI outcomes	[31], [14], [21], [23], [59], [30], [18]
TP53 + RB1 (± PTEN) co-alteration	Co-loss / combined alterations	Lineage plasticity / neuroendocrine-like features; ARSI resistance; highly aggressive biology	[21], [51], [41], [55]
PTEN	Deletion/loss	Poor response to AR-targeted therapy; aggressive course	[51], [60], [58] (poor ARPI rPFS)
HRR genes (BRCA1/2, ATM, etc.)	Pathogenic/truncating (germline & somatic)	PARP inhibitor benefit; greatest with BRCA1/2; mixed/limited benefit with non-BRCA HRR	[52], [46], [47], [14] (poor ARSI outcomes)
PALB2	Pathogenic + reversion mutations	Initial PARPi sensitivity; reversion mutations → acquired PARPi resistance	[39]
PMS2 / MMR	Pathogenic mutation / MSI-H	May benefit from checkpoint inhibitors (e.g., pembrolizumab)	[18], [22]
CDK12	Mutations / biallelic loss	High TMB → checkpoint inhibitor sensitivity; limited PARPi benefit	[36], [52]
PI3K pathway (PIK3CA, PTEN loss context)	Mutations/CN gain	AR-targeted therapy resistance; rationale for PI3K/AKT/mTOR combinations	[14], [21], [48]
Therapy-modality signal (Lu-PSMA)	AR, TP53, PTEN alterations	Poorer rPFS on ARPIs, but better rPFS with 177Lu-PSMA-617 vs changing ARPI	[58]
Platinum sensitivity signal	TP53 alterations	Poor OS overall, but better response to platinum chemo in AVPC context	[41]
NCOA2	CN gain / missense	Poor ARPI outcomes (no PSA responses when gain present)	[60]
