# Supplementary material for: Circulating Tumor DNA as a Biomarker for Precision Medicine in Prostate Cancer: A Systematic Review
Source: Int J Mol Sci. 2025 Nov 15;26(22):11049. doi: 10.3390/ijms262211049 (PMC12652532; doi:10.3390/ijms262211049)
Supplement: Supplementary file 1 [file ijms-26-11049-s001.zip › Supp_M_risk_of_bias_Tables_S2_S3_S4_S5_S6.pdf]

Table S2: A revised Cochrane risk-of-bias tool for randomized trials RoB 2

Study	Randomization Process	Deviations from Intended Interventions	Missing Outcome Data	Measurement of the Outcome	Selection of the Reported Result	Overall Risk
Annala et al., 2018	Low	Low	Low	Low	Some concerns	Some concerns
Carr et al., 2021	Low	Low	Low	Low	Some concerns	Some concerns

Table S3: QUIPS-Based Risk Of Bias Table

Studies	Study Participation	Study Attrition	PF Measurement	Outcome Measurement	Confounding	Statistical Analysis & Reporting	Overall Risk
Conteduca et al. 2017	Moderate	Low	Low	Low	Moderate	Low	Moderate
Torquato et al., 2019	Low	Low	Low	Low	Moderate	Low	Low
De Laere et al. 2019	Low	Low	Low	Moderate	Low	Low	Low
Moses et al., 2020	Moderate	Low	Low	Low	Serious	Moderate	Moderate
Kohli et al., 2020	Low	Low	Low	Low	Low	Moderate	Low
Reimers et al. 2020	Low	Moderate	Low	Low	Moderate	Low	Moderate
Dang et al. 2020	Low	Low	Low	Low	Moderate	Low	Low
Ledet et al. 2020	Moderate	Low	Low	Low	Moderate	Low	Moderate
Shaya et al. 2021	Moderate	Low	Low	Low	Moderate	Low	Moderate
Dong et al. 2021	Low	Low	Low	Low	Moderate	Low	Low
Lin et al. 2021	Low	Low	Low	Low	Moderate	Low	Low
Yu et al. 2021	Low	Low	Low	Low	Low	Low	Low

Fan et al. 2021	Moderate	Low	Low	Low	Moderate	Low	Moderate
Fettke et al. 2021	Low	Low	Low	Low	Moderate	Low	Low
Jayaram et al., 2021	Low	Low	Moderate	Low	Low	Low	Low
Pan et al. 2022	Moderate	Low	Low	Low	Moderate	Low	Moderate
Chi et al. 2023	Low	Low	Low	Low	Low	Low	Low
Du et al., 2023	Low	Low	Moderate	Moderate	Serious	Moderate	Moderate
Wang et al. 2023	Low	Low	Low	Low	Low	Low	Low
Dincman et al. 2024	Low	Low	Low	Low	Low	Low	Low

Table S4: NOS-Based Risk of Bias Table

Study	Selection Bias	Comparability	Outcome	Overall Risk
Wyatt et al. 2017	Low	Moderate	Moderate	Moderate
Chen et al. 2022	Moderate	Moderate	Moderate	Moderate
Loehr et al. 2022	Low	Low	Moderate	Low
Barata et al. 2022	Low	Low	Low	Low
Fei et al., 2023	Moderate	Serious	Moderate	Moderate

Table S5: ROBINS-I Risk of Bias Table

Study	Bias due t o confounding	Classification of interventions/ exposures	Selection of participants	Deviations from Interventions/ exposures	Bias due to Missing data	Outcome Measurement	Selective Reporting	Overall Risk
Torquato et al., 2019	Serious	Low	Moderate	Low	Moderate	Low	Moderate	Serious
Knutson et al. 2024	Moderate	Moderate	Low	Low	Moderate	Low	Moderate	Moderate

Table S6: Observational Cross-Sectional Study (JBI Tool)

Study	Defined Inclusion Criteria & Sample Selection	Subjects and Setting Described in Detail	Exposure Measurement (e.g. clinical factors)	Outcome Measurement (ctDNA positivity)	Identification of Confounding Factors	Strategies to Deal with Confounding	Outcome Assessment Reliability (e.g. follow-up)	Statistical Analysis Appropriateness	Overall Risk
Necchi et al. 2021	Low	Low	Low	Low	Serious	Serious	Low	Unclear	Moderate
Bang et al., 2023	Low	Low	Low	Low	Serious	Serious	Moderate	Low	Serious
