# Supplementary material for: Circulating Tumor DNA as a Biomarker for Precision Medicine in Prostate Cancer: A Systematic Review
Source: Int J Mol Sci. 2025 Nov 15;26(22):11049. doi: 10.3390/ijms262211049 (PMC12652532; doi:10.3390/ijms262211049)
Supplement: Supplementary file 1 [file ijms-26-11049-s001.zip › Supp_M_Table_S1.pdf]

Table S1: Characteristics of included studies and patients

Study	Type of study	Period of study	Type of PCa	N# of cases	Age median (years)	PSA (ng/mL)
Wyatt et al. 2017	Comparative cohort study (retrospective analysis)	before June 2017	mCRPC	45	70 (45–90)	70.2 (3.4–4478)
Conteduca et al. 2017	Multi-institutional observational biomarker study	2011 - 2016 (Primary cohort), 2015 - 2016 (PREMIERE trial)	CRPC	265 patients (171 in Primary cohort, 94 in PREMIERE trial)	75 (41-91	Primary cohort: Range 2-3150 ng/mL, PREMIERE trial: Range 3-4319 ng/mL
Wyatt et al. 2018	Biomarker analysis from an ongoing randomized phase II trial (abiraterone vs. enzalutamide)	N/A	mCRPC, chemotherapy-naïve	62	N/A	N/A
Annala et al. 2018	Prospective, randomized Phase II trial	November 2014 – October 2016	Treatment-naïve mCRPC	202	75.3 (range: 49.3–94.1)	36.1 (range: 1.7–2817)
Carneiro et al., 2018	Case Report with molecular analysis	N/A: longitudinal ctDNA monitoring.	SCCP: Aggressive neuroendocrine subtype May arise de novo or after transdifferentiation from prostate adenocarcinoma.	1	39	1.6 at diagnosis
Ma et al. 2018	Case report	2015–2017	mCRPC	1	67	>100 (diagnosis), = 644.3 before olaparib
De Laere et al., 2019	Prospective observational	March 2014 – April 2017	mCRPC	168	76 ± 7.7	36.92 (13.5–144.9)
Reimers et	Retrospective cohort	January 1, 1988 –	mCRPC	317	65 (58–74) for	20.9 (IQR 12.0–138.0)

al., 2019	study	March 16, 2018			CDK12-mutated patients	
Torquato S et al., 2019	Prospective cohort study	2014 – 2018	mCRPC	62	N/A	N/A
Dang et al. 2020	Prospective cohort study	November 2018 – November 2019	mPC including mCRPC	40	69	N/A
Fan et al. 2020	Retrospective cohort study	February 2018 – November 2019	CSPC and mCRPC	396 PCa: 213 CSPC (131 nmCSPC, 82 de novo mCSPC) 183 mCRPC	CSPC: 68 years De novo mCSPC: 68.5 years mCRPC: 66 years	CSPC: 52.7% had PSA <10 ng/mL mCRPC: 30.1% had PSA >100 ng/mL
Ledet et al., 2020	Retrospective cohort study	July 2, 2014 – August 15, 2017	Advanced prostate cancer (CRPC)	892	70 years (41–93)	N/A
Kohli et al., 2020	Prospective longitudinal cohort study	September 2009 – March 2014 (Mayo Clinic cohort) 2016 – 2018 (Monash University cohort)	mHSPC, mCRPC	250	mHSPC cohort: 66 years (range 45–90) mCRPC cohort: 72 years (range 50–92)	Untreated mHSPC: 6.35 ng/mL (IQR: 1.0–28.3) Clinical mCRPC: 16.7 ng/mL (IQR: 4.5–60.5)
Moses et al., 2020	Retrospective cohort study	May 2016 – April 2019	mCRPC	33	73 (60–88)	29.3 (0.04–845)
Goodall et al., 2020	Randomized Phase II Clinical Trial (NCT01485861)	N/A	mCRPC	216	N/A	N/A
Barata et al. 2021	Retrospective multi-institutional case series	September 2018 to April 2020	mCRPC	14	69 (55–88)	29.3
Ravindranathan et al., 2021	Case Series	N/A	mCRPC	2	Case1: 51; Case2: 81	Case 1: Initial PSA = 353 ng/mL, at pembrolizumab initiation = 39.90 ng/mL, undetectable after treatment Case 2: Initial PSA = 11 ng/mL, before pembrolizumab = 86.83 ng/mL, reduced

						to 0.11 ng/mL after treatment
Carr et al. 2021	Randomized, double-blind, placebo-controlled, Phase II trial	Trial: NCT01972218	mCRPC	142 randomized patients	N/A	N/A
Necchi et al., 2021	Retrospective observational study	N/A	Advanced prostate cancer, m and non m, CRPC and mCSPC	2462: Total ctDNA (liquid biopsy) samples. 1294 (770 primary tumors, 127 bone metastases, 34 liver, 25 lung, 7 brain, 205 lymph node, and 126 soft tissue samples)	Median age across different metastatic sites ranged from 64–75 years. Liquid biopsy: Median age 74 years (range 42–89 years).	N/A
Jayaram et al., 2021	Prospective biomarker analysis within a phase II clinical trial (NCT01867710)	June 2013 – October 2014	mCRPC	151	N/A	N/A
Shaya et al. 2021	Retrospective (cohort)	2014-2019	mCRPC	63	71 (46-94)	N/A
Agarwal et al. 2022	Exploratory biomarker analysis of ctDNA from a phase 3 randomized trial (TITAN)	N/A	mCSPC	114: Baseline ; End of study treatment (EOST: 129 patients)	N/A	N/A

Saad et al. 2022	Post hoc exploratory analysis of a phase 3 randomized trial (PROpel)	Baseline and subsequent PSA assessments with a second data cutoff on March 14, 2022	mCRPC	N/A	N/A	N/A
Sautois et al., 2022	Case Report	Patient diagnosed in 2006, mCRPC confirmed in 2016, treated with rucaparib from 2018 to 2020, deceased in 2021	mCRPC	1	63 at diagnosis	N/A
Hemenway et al. 2022	Case Report	2005–2020	mCRPC with secondary hairy-cell leukemia mCRPC with secondary hairy-cell leukemia	1	63	10.3 (at diagnosis)
Pan et al. 2022	Observational cohort study	Enrollment: April 2019 – March 2021; Median follow-up: 18.0 months (IQR 15.7–21.0)	mCRPC	106	M= 68 (IQR 63–73)	5.2 (IQR 3.3–14.3 ng/mL)
Chen et al. 2022	Prospective observational liquid biopsy profiling study	March 2017 – November 2018; Follow-up: March 2017 – December 2020	Prostate Cancer (both primary and metastatic) compared with benign prostatic hyperplasia (BPH)	33 PCa patients and 15 BPH patients	N/A (aggregated result not available, only individual)	N/A
Yuan et al. 2022	Case report	N/A	mCRPC	1	61	Initial serum total PSA (TPSA): 787 ng/mL<br>At best response during therapy: 8.02 ng/mL<br>At resistance stage: 601 ng/mL
Loehr et al.	Retrospective	Before May 5, 2020	mCRPC	100	72 (65–76)	87.1 (IQR: 29.8–311.2)

2022	analysis from TRITON2 clinical trial (Phase 2)					
Chi et al., 2023	Retrospective molecular profiling study	PROfound clinical trial (NCT02987543), screening phase	mCRPC	619 total screened, 503 ctDNA samples yielded results, 491 matched to tumor tissue	N/A	N/A
Dong et al., 2023	Retrospective and prospective cohort study	Prospective validation cohort: NCT03786848 (dates not explicitly mentioned) Clinical utility study: September 2009 – March 2014	mCRPC	Orthogonal validation cohort: 15 patients Clinical utility cohort: 52 patients	N/A	N/A
Fettke et al. 2023	Prospective cohort study	September 2016 - August 2018	mCRPC	91	73 (46-91)	28.6 (0.51-2719)
Wang et al. 2023	Retrospective cohort study	January 2018 – August 2021	Aggressive-Variant Prostate Cancer (AVPC)	63	65.84 ± 8.89	66.17 (IQR: 24.52–161.86)
Fei et al., 2023	Retrospective observational study	N/A	nmPCa	161	66 (61.5–70.5)	63 patients had 0–20 ng/mL, 47 had 20–100 ng/mL, and 20 had >100 ng/mL
Bang et al., 2023	Retrospective study	May 2021 – March 2023	mPC (mCRPC and mHSPC)	100	N/A	mCRPC cohort: 75.0 ng/mL mHSPC cohort: N/A
Du et al., 2023	Retrospective observational study	October 2019 – June 2020	mHSPC	66	65 (61–68)	64 (34.8–151.2)
Oya et al., 2023	Randomized, Double-Blind, Placebo-Controlled Phase III Clinical	N/A	mCRPC	796 (399 in the abiraterone + olaparib arm,	N/A	N/A

	Trial (NCT03732820)			397 in the placebo + abiraterone arm)		
Clarke et al., 2023	Phase 3 randomized controlled trial (RCT)	N/A	mCRPC	796	N/A	N/A
Knutson et al. 2024	Retrospective correlative analysis of plasma samples from a phase 3 randomized trial (Alliance A031201)	N/A	mCRPC	776 cfDNA specimens that passed quality control (from a larger trial cohort)	N/A	N/A
Kristiansen 2024 et a2024l. 2024	Randomised, outcome-adaptive, biomarker-driven trial comparing carboplatin versus standard-of-care (SOC) in mCRPC.	N/A	mCRPC	115	N/A	N/A
Lin et al. 2024	Clinical and Translational Research Study Retrospective analysis	June 2016 – February 2020	mCRPC	132 : mCRPC were analyzed for plasma lipidomic profiles.<br>77: ctDNA sequenced for AR gene aberrations	74 (Q1 = 67, Q3 = 80)	ctDNA cohort (n=77): Median 19 ng/mL (Q1 = 6, Q3 = 83)<br>Overall cohort (n=132): Median 28 ng/mL (Q1 = 9, Q3 = 66)
Yu et al. 2024	clinical and Translational Research Study	November 2017 – February 2020	mHSPC, mCRPC	56 (ctDNA analysis).<br>18 of these	N/A	N/A

	Retrospective cohort study			also solid tissue.		
Dincman et al., 2024	Single-institution, retrospective cohort study	March 2015 – March 2020	mCRPC	155 (130 at least 1 GA; 83 mCRPC for survival analysis)	71 (46–91)	46.2 (0.1–6000)
De Bono et al. 2024	Exploratory analysis from a randomized trial (PSMAfore, NCT04689828)	cutoff as of June 2023	MCRPC, taxane-naïve	468, 360 samples were collected, 255 passed quality control, and 156 had ctDNA fraction >1%.	N/A	N/A

PCa, prostate cancer; mCRPC, metastatic castration-resistant prostate cancer; CRPC, castration-resistant prostate cancer; mHSPC, metastatic hormone-sensitive prostate cancer; nmCSPC, non-metastatic castration-sensitive prostate cancer; de novo mCSPC, de novo metastatic castration-sensitive prostate cancer; SCCP, small-cell carcinoma of the prostate; mPC, metastatic prostate cancer; nmPCa, non-metastatic prostate cancer; AVPC, aggressive-variant prostate cancer; cfDNA, cell-free DNA; ctDNA, circulating tumor DNA; PSA, prostate-specific antigen; N/A, not available; IQR, interquartile range; SOC, standard of care; RCT, randomized controlled trial.
