# Supplementary material for: Circulating Tumor DNA as a Biomarker for Precision Medicine in Prostate Cancer: A Systematic Review
Source: Int J Mol Sci. 2025 Nov 15;26(22):11049. doi: 10.3390/ijms262211049 (PMC12652532; doi:10.3390/ijms262211049)
Supplement: Supplementary file 1 [file ijms-26-11049-s001.zip › Supp_M_Table_S7.pdf]

Table S7 : Specimens and Genetic Alterations

Reference	Sample Type	ctDNA detection method	Gene	Somatic/Germline	Type of alteration	Site
Agarwal et al. 2022	Plasma	NGS	AR	Somatic	CNG (amplification) + (activation/inactivation)	N/A
			TP53		Inactivation (SNV/deletion)	N/A
			HRR Pathway*		Inactivation (loss-of-function mutations)	N/A
			PTEN		Inactivation	N/A
			RB1		Inactivation	N/A
			PIK3CA		Activation	N/A
Annala et al. 2018	Plasma	NGS	BRCA2/ATM	germline & somatic	Truncating mutation	N/A
			BRCA2/ATM		Monoallelic deletion	N/A
			MSH2		Frameshift mutation	N/A
			MLH1		Frameshift mutation	N/A
			MSH6		Loss	N/A
			TP53		Mutation/Deletion	N/A
			RB1		Deletion/Defect	N/A
			AR		Amplification	N/A
			AR		Missense in LBD	H875Y, W742*
			AR	Somatic	Genomic structural rearrangement	11.4-kb deletion from 23 bp downstream of exon 4 to within exon 7 (truncates LBD)
			SPOP		Mutation	N/A

			PI3K Pathway		Defect	N/A
			WNT Pathway		Defect	N/A
Bang et al., 2023	Plasma	NGS	AR	Somatic	Various mutations	N/A
			TP53	Somatic	Various mutations	N/A
			RB1	Somatic	Various mutations	N/A
			PTEN	Somatic	Deletions/mutations	N/A
			APC	Somatic	Mutations	N/A
			BRCA2	Germline	Pathogenic/Likely Pathogenic Mutation	N/A
			BRCA1	Germline	Pathogenic/Likely Pathogenic Mutation	N/A
			ATM	Germline	Pathogenic Mutation	N/A
			CHECK2	Germline	Pathogenic Mutation	N/A
			BRIP1	Germline	Pathogenic Mutation	N/A
			FANCA	Germline	Pathogenic Mutation	N/A
			MSH2	Germline	Pathogenic Mutation	N/A
			MSH6	Germline	Pathogenic Mutation	N/A
Barata et al. 2021	Plasma	NGS	MSI-H	Somatic	Microsatellite instability high	90 microsatellite loci
			TP53	Somatic	N/A	N/A
			AR	Somatic	N/A	N/A
			ARID1A	Somatic	N/A	N/A
			PTEN	Somatic	N/A	N/A
			BRCA1	Somatic	Pathogenic alteration	N/A
			BRCA2	Somatic	Pathogenic alteration	N/A

			PIK3CA	Somatic	N/A	N/A
			FGFR1/2	Somatic	N/A	N/A
			ATM	Somatic	N/A	N/A
Carneiro et al., 2018	Plasma	NGS	ALK	Somatic	Activating point mutation	F1174C
			ATM	Germline	Missense	R2060H
			TP53	Somatic	Missense	R248Q
			ARID1A	Somatic	nonsense	Q268*
			FAS	Somatic	Frameshift	L12FS*12
			AXIN1	Somatic	N/A	N/A
			FAT1	Somatic	N/A	N/A
			PIK3R2	Somatic	N/A	N/A
			SMARCA4	Somatic	N/A	N/A
			KDM5A	Somatic	N/A	N/A
Carr et al. 2021	Plasma	NGS	BRCA2	Somatic & Germline	Frameshift, nonsense, Copy number deletions	N/A
			ATM	Somatic & Germline	Frameshift, Copy number deletions	N/A
			CDK12	Somatic	point mutation	N/A
			CHEK2	Germline	Missense	N/A
			PALB2	Germline	Missense	N/A
			PPP2R2A	Somatic	Copy number loss	N/A
Chen et al. 2022	Plasma and urine	NGS	TP53	Somatic	Mutation	N/A
			AR	Somatic	Mutation/Variant	N/A
			ATM	Somatic	Mutation	N/A

			MYC	Somatic	mutation and/or amplification	N/A
			APC	Somatic	Mutation	N/A
			CTNNB1	Somatic	Mutation	N/A
			SPOP	Somatic	Mutation	N/A
			BRAF	Somatic	Mutation	N/A
			ESR1	Somatic	Mutation	N/A
			CDH1	Somatic	Mutation	N/A
			PIK3CA	Somatic	point mutation in BPH	p.Arg108His (hotspot mutation)
			GNAS	Somatic	Mutation in BPH	N/A
			VHL	Somatic	Mutation in BPH	N/A
			CDK4	Somatic	Mutation in BPH	N/A
			EGFR	Somatic	Mutation in BPH	N/A
			NF1	Somatic	Mutation in BPH	N/A
			RB1	Somatic	Mutation in BPH	N/A
			SMAD4	Somatic	Mutation in BPH	N/A
			KMT2D	Somatic	Mutation	N/A
			FGFR2	Somatic	Mutation	N/A
			ARID1A	Somatic	Mutation	N/A
			ARAF	Somatic	Mutation	N/A
Chi et al., 2023	Plasma	NGS	BRCA1	N/A	Frameshift, nonsense	N/A
			BRCA1	N/A	Large rearrangements	N/A
			BRCA1	N/A	Homozygous deletions	N/A

			BRCA2	N/A	Frameshift, nonsense	N/A
			BRCA2	N/A	Large rearrangements	N/A
			BRCA2	N/A	Homozygous deletions	N/A
			ATM	N/A	Missense mutations	N/A
			ATM	N/A	Splice-site mutations	N/A
			ATM	N/A	Large rearrangements	N/A
Clarke et al., 2023	Plasma	NGS	HRR	N/A	N/A	N/A
			BRCA (BRCA1/2)	N/A	N/A	N/A
Conteduca et al. 2017	Plasma	ddPCR and NGS	AR	Somatic	CNV- Amplification	N/A
			AR	Somatic	Missense	c.2105T>A (p.L702H)
			AR	Somatic	Missense	c.2632A>G (p.T878A)
			AR	Somatic	Missense	c.2629T>C (p.F877L)
Dang et al. 2020	Plasma	NGS	AR	Somatic	Enhancer Amplification (40%)	Upstream of AR gene
			AR	Somatic	Gene Body Amplification	N/A
			TP53	Somatic	Copy Number Loss (15%)	N/A
			TP53	Somatic	Nonsynonymous SNV (13%)	N/A
			PTEN	Somatic	Copy Number Loss (15%)	N/A
			TMPRSS2-ERG	Somatic	Gene Fusion (13%)	N/A
De Bono et al. 2024	Plasma	NGS	AR	Somatic	Alteration (unspecified)	N/A
			TP53	Somatic	Alteration (unspecified)	N/A

			PTEN	Somatic	Alteration (unspecified)	N/A
De Laere et al., 2019	Plasma	NGS	TP53	Somatic	Mutation, Deletion	N/A
			AR	Somatic	Amplification, Structural Rearrangement	N/A
			AR	Somatic	Mutation	L702H
					Mutation	H875Y
Dincman et al., 2024	Plasma	NGS	AR	Somatic	Amplification	PCN range: 1.2–35.4 (median: 2.03)
			MYC	Somatic	Amplification	PCN range: Not specified
			BRAF	Somatic	Amplification	7q34
			CDK6	Somatic	Amplification	7q21.2
			PIK3CA	Somatic	Amplification	N/A
			MET	Somatic	Amplification	7q31.2
			FGFR1	Somatic	Amplification	N/A
			EGFR	Somatic	Amplification	N/A
			RAF1	Somatic	Amplification	N/A
			TP53	Somatic	Mutation	N/A
			APC	Somatic	Mutation	N/A
			BRCA2	Somatic	Mutation	N/A
			ATM	Somatic	Mutation	N/A
Dong et al., 2023	Plasma	NGS	PTEN	Somatic	Copy number loss	N/A
			RB1	Somatic	Copy number loss	N/A
			TP53	Somatic	Copy number loss	N/A
			MYC	Somatic	Copy number gain (amplification)	N/A
			AR	Somatic	Copy number gain (amplification)	N/A

Du et al., 2023	Plasma	NGS	CDK12	Somatic	Mutation	N/A
			AR	Somatic	Mutation	N/A
			NCOR2	Somatic	Mutation	N/A
			ATR	Somatic	Mutation	N/A
			BRCA2	Somatic	Mutation	N/A
			ATM	Somatic	Mutation	N/A
			BRCA2	Germline	Mutation	N/A
Fan et al. 2020	Plasma	NGS	AR	Somatic	Amplification, mutations	N/A
			CDK12	Somatic	Mutations	N/A
			FOXA1	Somatic	Mutations	N/A
			BRCA2	Somatic & Germline	Germline & somatic mutations	N/A
			BRCA1	Somatic & Germline	Germline & somatic mutations	N/A
			ATM	Somatic & Germline	Germline & somatic mutations	N/A
			PALB2	Germline	Mutation	N/A
			TP53	Somatic	Copy number loss, mutation	N/A
			RB1	Somatic	Mutation	N/A
			PTEN	Somatic	Copy number loss	N/A
Fei et al., 2023	Plasma	NGS	NCOR2	Somatic	Deleterious mutations	N/A
			BRCA2	Somatic	Deleterious mutations	N/A
			ATR	Somatic	Deleterious mutations	N/A
			CDK12	Somatic	Deleterious mutations	N/A
Fettke et al. 2023	Plasma	NGS	NCOA2	Somatic	Copy number gain	8q12

			NCOA2	Somatic	Missense	N/A
			NCOA2	Germline	Missense	N/A
			AR	Somatic	Copy number gain	Xq12
			AR	Somatic	Missense	L702H
			AR	Somatic	Missense	V716M
			PTEN	Somatic	Deletion	10q23
			RB1	Somatic	Deletion	13q14
			TP53	Somatic	Missense	R248Q
Goodall et al., 2020	Plasma	NGS	TP53	Somatic	Mutation	N/A
			AR	Somatic	Mutation	N/A
			FOXA	Somatic	Mutation	N/A
			PTEN	Somatic	Mutation	N/A
			PI3K/AKT pathway genes	Somatic	Mutation	N/A
Hemenway et al. 2022	Plasma	NGS	CHEK2	Germline	Frameshift	T367fs*15
			BRAF	Somatic	Point mutation	BRAF V600E
			NF1	Somatic	Point mutation	N/A
			JAK2	Somatic	Point mutation	N/A
			RB1	Somatic	Point mutation	N/A
			AR	Somatic	Amplification	CNG: median copies ~7.1
			AR LBD	Somatic	Missense	H875Y
Jayaram et al., 2021	Plasma	NGS	TP53	Somatic	Mutation, Deletion	N/A
			RB1	Somatic	Deletion	N/A

			PTEN	Somatic	Mutation, Deletion	N/A
			AR	Somatic	Copy Number Gain	N/A
			PIK3CA	Somatic	Mutation, CNG	N/A
			BRCA2	Somatic	Biallelic Alteration	N/A
			ATM	Somatic	Biallelic Alteration	N/A
			CHEK2	Somatic	Biallelic Alteration	N/A
			FANCA	Somatic	Biallelic Alteration	N/A
			HDAC2	Somatic	Biallelic Alteration	N/A
Knutson et al. 2024	Plasma	NGS	AR	Somatic	CNG, Structural rearrangements (AR-GSRs) (and infrequent point mutations)	LBD
			TP53	Somatic	Point mutations and CNL	N/A
			PTEN	Somatic	CNL	N/A
			RB1	Somatic	CNL	N/A
			MYC	Somatic	CNG	N/A
			MYCN	Somatic	CNG	N/A
Kohli et al., 2020	Plasma	NGS	AR	Somatic	Amplifications, SNVs	T742L, T742C, V716M, T878A, L702H, H875Y
			TP53	Somatic	Missense mutations, deletions	N/A
			RB1	Somatic	Copy number loss (deletion)	N/A
			ATM	Somatic & Germline	Deleterious mutations	N/A
			BRCA1	Somatic & Germline	Deleterious mutations	N/A
			BRCA2	Somatic & Germline	Deleterious mutations	N/A
			CHEK2	Somatic & Germline	Deleterious mutations	N/A

			CDK6	Somatic	Amplifications	N/A
			CDH1	Somatic	Deletions	N/A
			EGFR	Somatic	Amplifications	N/A
			MYC	Somatic	Amplifications	N/A
			BRAF	Somatic	Amplifications	N/A
Kristiansen et al. 2024	Plasma	N/A	TP53	Somatic	Mutation (inactivation/loss-of-function)	Not specified (N/A)
			DRD (including BRCA1/2)	Somatic	Mutation/inactivation (DNA repair deficiency)	Not specified (N/A)
Ledet et al., 2020	Plasma	NGS	AR	Somatic	Amplification	N/A
			AR	Somatic	Truncating mutation	N/A
			AR	Somatic	Nonsynonymous	N/A
			AR	Somatic	Missense	L702H
			AR	Somatic	Missense	T878A
			AR	Somatic	Missense	H875Y
			AR	Somatic	Missense	W742C
			AR	Somatic	Missense	W742L
			AR	Somatic	Missense	F877L
			AR	Somatic	Missense	T878S
			AR	Somatic	Missense	V716M
			AR	Somatic	Missense	D891H
			AR	Somatic	Missense	M750V
			AR	Somatic	Missense	M750T

			AR	Somatic	Missense	S889G
			TP53	Somatic	point mutation	N/A
			MYC	Somatic	CNV-Amplification	N/A
			BRAF	Somatic	point mutation	N/A
			PIK3CA	Somatic	point mutation	N/A
			MET	Somatic	point mutation	N/A
			CDK6	Somatic	CNV-Amplification	N/A
			EGFR	Somatic	point mutation	N/A
			FGFR1	Somatic	point mutation	N/A
			APC	Somatic	point mutation	N/A
			BRCA2	Somatic	point mutation	N/A
			BRCA1	Somatic	point mutation	N/A
			ATM	Somatic	point mutation	N/A
Lin et al. 2024	Plasma	NGS	AR	Somatic	CNG	N/A
			AR	Somatic	Missense	N/A
Loehr et al. 2022	Plasma	NGS	BRCA1	Somatic & Germline	Reversion mutations: indels, point mutations	N/A
			BRCA2	Somatic & Germline	Reversion mutations: indels, point mutations	N/A
			BRCA2	Germline	Deletion→ frameshift	c.6174delT (S1982fs)
			Other	N/A	Not explicitly listed for other genes	N/A
Ma et al. 2018	Plasma	NGS	BRCA2	Germline	nonsense	c.G5281T, p.G1761X

			PIK3CA	Somatic	Missense	c.C1636A, p.Q546K
			TP53	Somatic	Fusion	DISCIFP1 fusion
			AR	Somatic	CNV- Amplification	N/A
			RB1	Somatic	CNV-Loss/Deletion	N/A
			NKX2-1	Somatic	Missense	c.C512T, p.A171V
			ERBB4	Somatic	Missense	c.C842G, p.A281G
			RUNX1	Somatic	In-frame deletion	c.1408_1437del
			NF1	Somatic	Missense	c.G520A, p.V174I
			MET	Somatic	Intron variant	N/A
			FGFR4	Somatic	Missense	c.C1480A, p.P494T
			TET2	Somatic	Frameshift	c.2838delT, p.T946fs
Moses et al., 2020	Plasma	NGS	AR	Somatic	Amplifications	N/A
			AR	Somatic	Missense mutations	W742C, T878A, L702H, M896V, V716M
			TP53	Somatic	Various mutations	N/A
			BRCA2	Somatic	Frameshift mutations	C2363fs, W2970*, L2357fs
			BRCA1	Somatic	Missense mutation	M1?
			ATM	Somatic	Missense mutation	R3008C
			BRCA2	Germline	Frameshift mutations	V1486Nfs, L2357Vfs
			BRCA1	Germline	Initiator codon mutation	M1?
			ATM	Germline	Frameshift mutation	L762Vfs
			HOXB13	Germline	Missense mutation	G84E
			PMS2	Germline	Mutation	N/A

			MUTYH	Germline	Mutation	N/A
Necchi et al., 2021	Plasma	NGS	AR	Somatic	Amplifications, Short Variants, Rearrangements	N/A
			TP53	Somatic	Mutations	N/A
			PTEN	Somatic	Deletions, Short Variants	N/A
			BRCA2	Somatic	Deletions, Short Variants	N/A
			ATM	Somatic	Mutations	N/A
			RB1	Somatic	Deletions, Mutations	N/A
			PIK3CA	Somatic	Mutations	N/A
			CDK12	Somatic	Mutations	N/A
			BRAF	Somatic	Mutations	N/A
			ERBB2	Somatic	Amplifications, Short Variants	N/A
			MSI-high	Somatic	Microsatellite Instability	N/A
			TMB ≥10 mut/Mb	Somatic	High Tumor Mutational Burden	N/A
Oya et al., 2023	Plasma	NGS	HRR-related genes **	Somatic/Germline	Point Mutation	N/A
			BRCA1/BRCA2	Somatic/Germline	Point Mutation	N/A
Pan et al. 2022	Plasma	NGS	CDK12	somatic &/germline	Deleterious point mutation	N/A
			BRCA2	somatic &/germline	Deleterious mutation	N/A
			CHEK2	somatic &/germline	Deleterious mutation	N/A
			BRIP1	somatic &/germline	Deleterious mutation	N/A
			ATM	somatic &/germline	Deleterious mutation	N/A

			PTEN	N/A	Loss of expression	Protein loss indicative of PTEN loss
Ravindranathan et al., 2021	Plasma	NGS	MSI	Somatic	High MSI-H status	N/A
Reimers et al., 2019	Plasma	NGS	CDK12	Mostly somatic	Biallelic loss	Not specified, mutations in RS, PRM, or kinase domains
			BRCA1	Germline (some cases)	Deletion, point mutations, frameshift mutations	N/A
			BRCA2	Germline (majority)	Deletion, point mutations, frameshift mutations	N/A
			ATM	Germline (some cases)	Deletion, point mutations, frameshift mutations	N/A
			TP53	N/A	Point mutations	N/A
Saad et al. 2022	Plasma	NGS	AR	Somatic	CNG (amplification)	N/A
			AR	Somatic	Point mutation	N/A
			TP53	Somatic	Inactivation (loss-of-function mutation)	N/A
			BRCA2	Somatic	Inactivation (loss-of-function)	N/A
Sautois et al., 2022	Plasma	NGS	RAD51B	Somatic	Truncating rearrangement (fusion with ACTN1)	Deletion of exons 3–11
			RB1	Somatic	Rearrangement	N/A
			TP53	Somatic	2 Mutations	N/A
			TMPRSS2-ERG	Somatic	Fusion	N/A
Shaya et al. 2021	N/A	NGS	AR, TP53, PIK3CA, ATM, BRCA1, BRCA2, CDK12, PMS2	Somatic	point mutations	N/A
			AR, PTEN	N/A	CNA: AR: Amplification PTEN Loss	N/A

			RB		RB1 Loss	
Torquato S et al., 2019	Plasma	NGS	AR	Somatic	Ligand-binding domain mutations	N/A
			AR	Somatic	Copy number (CN) gain	N/A
			TP53	Somatic	Various mutations	N/A
			RB1	Somatic	Various mutations	N/A
			PTEN	Somatic	Deletions/mutations	N/A
			PIK3CA	Somatic	Copy number gain/mutations	N/A
			APC	Somatic	Mutations	N/A
			BRCA2	Germline	Pathogenic Mutation	N/A
			BRCA1	Germline	Pathogenic Mutation	N/A
			ATM	Germline	Pathogenic Mutation	N/A
Wang et al. 2023	Plasma	NGS	TP53	Somatic	Point mutations, deletions	N/A
			RB1	Somatic	CNV-Loss/Deletion	N/A
			PTEN	Somatic	CNV-Loss/Deletion	N/A
			AR	Somatic	CNV-Amplification	N/A
			FOXA1	Somatic	point mutation	N/A
			CDK12	Somatic	point mutation	N/A
			PIK3CA	Somatic	point mutation	N/A
			MYCN	Somatic	CNG	N/A
			CYLD	Somatic	point mutation	N/A
			APC	Somatic	point mutation	N/A

Wyatt et al. 2017	Plasma	NGS	AR	Somatic	Amplifications	N/A
			SPOP	Somatic	Point mutations	N/A
			TP53	Somatic	Inactivating mutations	N/A
			PTEN	Somatic	Inactivating mutations	N/A
			RB1	Somatic	Inactivating mutations	N/A
			APC	Somatic	Inactivating mutations	N/A
			CDKN1B	Somatic	Inactivating mutations	N/A
			BRCA2	Somatic	Inactivating mutations	N/A
			PIK3R1	Somatic	Inactivating mutations	N/A
			ATM	Somatic	CNA	N/A
			PIK3CA	Somatic	CNA	N/A
			PIK3CB	Somatic	CNA	N/A
Wyatt et al. 2016	Plasma	NGS	AR	Somatic	CNG (amplification)	N/A
			AR	Somatic	Point mutation	N/A
			TP53	Somatic	Inactivation (loss-of-function; mutation/deletion)	N/A
			BRCA2	Somatic	Inactivation (loss-of-function)	N/A
Yu et al. 2024	Plasma	NGS	TP53	Somatic	Mutation	p.Leu114Ter
			PIK3CA	Somatic	Mutation	N/A
			TMPRSS2-ERG	Somatic	Fusion	E4 (exon 4 fusion)
			MYC	Somatic	Amplification	N/A
			FGFR3	Somatic	Amplification	N/A
			MET	Somatic	Amplification	N/A

	HRAS	Somatic	Mutation	N/A
	CCND1	Somatic	Amplification	N/A
	IDH1	Somatic	Mutation	N/A
	RAD50	Germline	Splice	NM_005732.3:c.3036+1G>A
	BRCA1	Germline	nonsense	p.Ser1007Ter
Yuan et al. 2022	PALB2	Germline	nonsense	c.751C>T (p.Q251*), Exon 4
	PALB2	Somatic	Indel	c.751_752delCAinsTT (p.Q251L) Exon 4
	PALB2	Somatic	Indel	c.751_753delCAGinsTAC (p.Q251Y) Exon 4
	PTEN	Somatic	Frameshift deletion	c.136_137del (p.Y46Qfs*5) Exon 2
	AR	Somatic	Copy number gain	Amplification
	CHD1	Somatic	Copy number loss	Loss/Deletion
	FGFR1	Somatic	Copy number gain	Amplification
	TP53	Somatic	Deletion	c.665_672*11del Exon 6-IVS6
	NOTCH2	Somatic	Splice site mutation	c.5311-1G>A IVS29
	PIK3C2G	Somatic	Missense	c.2143A>G (p.R715G) Exon 15
	LHCGR	Somatic	Missense	c.143C>T (p.T48M) Exon 1
	CDC25C	Somatic	Indel	c.1150_1151delGGinsCC (p.G384P) Exon 12
	FLT4	Somatic	Missense	c.376G>A (p.A126T) Exon 3

*: HRR pathway group: BRCA1, BRCA2, FANCA, BRIP1, HDAC2, CDK12, ATM, PALB2, and CHEK2.

**: (ATM, BRCA1, BRCA2, BARD1, BRIP1, CDK12, CHEK1, CHEK2, FANCL, PALB2, RAD51B, RAD51C, RAD51D, RAD54L)
