# Supplementary material for: Circulating Tumor DNA as a Biomarker for Precision Medicine in Prostate Cancer: A Systematic Review
Source: Int J Mol Sci. 2025 Nov 15;26(22):11049. doi: 10.3390/ijms262211049 (PMC12652532; doi:10.3390/ijms262211049)
Supplement: Supplementary file 1 [file ijms-26-11049-s001.zip › Supp_M_Table_S8.pdf]

Table S8: Association of ctDNA detected genomic alterations with clinical significance

Reference	Genetic Alteration	Clinical significance OS/PFS	Clinical outcome/Therapy associations
Shaya et al. 2021	AR alterations (mutations/amplifications)	Associated with inferior OS (HR = 2.22, P = 0.014 in univariate analysis)<br>Not significant in multivariate model (HR = 0.54, *P* = 0.22)	Commonly detected. Associated with resistance to ARSI
	>1 alteration detected	Strongly associated with worse OS:<br>Median OS: 8.8 vs. 26.1 months; HR = 7.0, *P* < 0.001 (univariate); HR = 16.7, *P* = 0.001 (multivariate)	Suggests high genomic instability; associated with disease progression
	High maximum allelic fraction (mAF >6.4%)	Associated with worse OS: 7.6 vs. 24.7 months; HR = 3.9, *P* < 0.001 (univariate). Not significant in multivariate analysis	Reflects high tumor burden
	Tumor suppressor genes (TP53, PTEN, RB1)	Trend toward inferior OS (HR = 1.71, *P* = 0.094), not statistically significant	Frequently altered; implications in disease aggressiveness and ARSI resistance.
	HRR pathway alterations (BRCA1/2, ATM, CDK12)	Actionable	Predictive for benefit from PARP inhibitors
	PMS2 (mismatch repair)	Actionable	May benefit from checkpoint inhibitors (e.g. pembrolizumab)
Clarke et al., 2023	HRR (BRCA1/2, ATM, CDK12, CHEK2)	improved OS	Reported association with improved OS in abiraterone + olaparib-treated patients
	BRCA (BRCA1/2)	Best OS HR 0.42	greatest survival benefit with olaparib + abiraterone
Conteduca et al. 2017	AR CNV: Amplification	Worse OS in both chemo-naïve and post-docetaxel patients	Associated with resistance to enzalutamide/abiraterone
	AR c.2105T>A (p.Leu702His)	Worse OS (HR 3.26, p = 0.004)	resistance to abiraterone (via prednisone activation)
	AR c.2632A>G (p.Thr878Ala)	Worse OS (HR 3.26, p = 0.004)	resistance to abiraterone<br>Activates AR signaling via progesterone<br>Worse OS (HR 3.26, p = 0.004) and PFS
	AR c.2629T>C (p.Phe877Leu)	N/A	It was tested for in patients receiving enzalutamide, but was not found in plasma ctDNA
Dang et al. 2020	AR Enhancer Amplification (40%)	Poor OS	Predicts resistance to AR-directed therapy,

	AR Gene Body Amplification	Poor OS	Predicts resistance to AR-directed therapy.
	TP53 Copy Number Loss (15%)	Worse OS	Associated with treatment resistance in prostate cancer.
	TP 53 Nonsynonymous SNV (13%)	Worse OS	clinical resistance to androgen receptor (AR)-directed therapy.
	PTEN Copy Number Loss (15%)	Not statistically evaluated	Linked to more aggressive disease and resistance to targeted therapies
	TMPRSS2-ERG gene fusion	N/A	Common in prostate cancer, role in resistance not detailed in this study
Agarwal et al. 2022	AR: Amplification and other aberrations (activation/inactivation)	Baseline: HR ~6.7; EOST: HR ~1.7 — worse OS	Significantly associated with poor OS
	TP53: Inactivation (SNV/deletion; unspecified details)	Worse OS in subsequent therapy (HR ~3.0, $p < 0.01$)	Poor OS in chemo-treated patients
	HRR genes: Inactivation (loss-of-function mutations)	Increased from baseline to EOST; not significant ($p = 0.5$)	No significant association with OS
	PTEN: Inactivation (likely copy number loss)	poor OS observed but not statistically significant ($p = 0.14$)	Poor OS trend observed
	RB1: Inactivation (likely copy number loss)	OS not statistically significant ($p = 0.11$)	Association with OS evaluated
	PIK3CA: Activation (likely due to SNV and/or amplification)	Worse OS (HR ~3.7 univariate; HR ~2.2 at EOST, $p < 0.001$)	Associated with poor OS in chemo-treated patients
Dong et al. 2021	PTEN Copy number loss	Significantly worse survival: median OS 18.6 vs. 37.4 months ($p = 0.002$)	Linked to aggressive disease and poor response to standard therapies.
	RB1 Copy number loss	shorter overall survival ($p = 2.2 \times 10^{-6}$)	alone led to a shorter OS; key driver of neuroendocrine-like transformation and androgen deprivation resistance.
	TP53 Copy number loss	Significantly reduced OS: median OS 14.7 months	Promotes tumor plasticity and treatment resistance, yielding highly aggressive phenotype
	MYC Copy number gain (amplification)	N/A	Common in prostate cancer; role in resistance not detailed in this study
	AR Copy number gain (amplification)	N/A	Associated with AR-directed therapy resistance, though not specifically quantified here
Lin et al. 2021	AR Copy Number Gains	Shorter OS: HR = 3.62, ($p < 0.001$). Shorter rPFS: median 3.9 months (vs 8.3 months in those with only one risk factor and 17.7 months in those with neither),(HR = 2.10, $p = 0.01$)	Associated with resistance to AR signaling inhibitors (ARSI) (enzalutamide/abiraterone); shorter treatment duration
	AR Missense (12 patients (16%))	Poorer survival outcomes (exact HR not specified, but significant)	Linked to worse prognosis on ARSI
Fettke et al. 2021	NCOA2: Copy number gain (8q12)	Worse OS. Median OS 10.1 vs. 18.3 months; $p = .004$; median PFS 2.7 vs. 4.3 months; $p = .005$	No patients with NCOA2 gain attained PSA response to ARPI (0% vs. 64%); marker of poor outcome on ARPI

	NCOA2: missense	shorter OS: HR~ 3.0; p = 0.02	Indicates more aggressive disease; poorer prognosis
	AR: Copy number gain (Xq12)	worse PFS, particularly in ARPI-treated patients (HR ~3.1; p = 0.003)	Predicts resistance to AR pathway inhibitors
	AR: missense mutations L702H, V716M	No independent link to OS	May contribute to resistance mechanisms but not independently prognostic here
	PTEN: Deletion (10q23)	shorter survival times	Associated with aggressive disease and likely poorer response
	RB1: Deletion (13q14)	shorter survival times ; lower PSA response	Contributes to aggressive phenotype and treatment resistance
	TP53: Missense mutation R248Q	shorter survival times ; lower PSA response	Indicates treatment resistance and poorer prognosis
Wang et al. 2023	TP53 Point mutations/deletions	Worse OS; better response to platinum-based chemotherapy	better response to platinum-based chemotherapy ; indicates aggressive disease
	RB1 CNV-Loss/Deletion	shorter OS	poor prognosis; loss correlates with aggressive disease progression and shorter Survival
	PTEN CNV-Loss/Deletion	poor prognosis	Potential resistance to AR-targeted therapy; indicates aggressive phenotype
	AR CNV-Amplification	N/A	Frequent in AVPC but not predictive of platinum response
	FOXA1 point mutation	N/A	Implicated in AR signaling alterations; no specified clinical impact in this cohort
	CDK12 point mutation	N/A	Potential link to immunotherapy response, but not evaluated here
	PIK3CA	N/A	Potential driver of resistance to AR-targeted therapy
	MYCN	N/A	Implicated in AVPC progression, but not correlated with chemotherapy response
	CYLD	N/A	Implicated in AVPC progression, but no specific clinical significance reported
	APC	N/A	Potential tumor suppressor loss, no direct clinical correlation shown
Dincman et al. 2024	AR amplification	Better OS when isolated (median 19.3 vs. 8.9 months if with other GAs)	AR-only amplification indicates less aggressive profile vs. co-occurring GAs
	MYC amplification	N/A	Poor prognosis; often co-occurs with TP53 mutations
	BRAF amplification (7q34)	N/A	Associated with liver/lung metastases; co-amplifies with CDK6, MET
	CDK6 amplification (7q21.2)	N/A	Linked to liver/lung metastases; co-amplifies with BRAF
	PIK3CA amplification	poor OS (median 5.9 vs. 16.0 months for other GAs)	Strong indicator of poor survival; linked to increased visceral metastases
	MET amplification (7q31.2)	N/A	Co-amplified with BRAF and CDK6; associated with more aggressive disease
	FGFR1 amplification	N/A	Associated with increased liver/lung metastases

	EGFR amplification	N/A	May contribute to tumor aggressiveness
	RAF1 amplification	N/A	Possible impact on prognosis, but less frequent
	TP53 mutation	N/A	Strongly linked to multiple GAs, particularly MYC and BRAF; indicates aggressive, heterogeneous disease
	APC mutation	N/A	Rare; minimal co-occurrence with AR/MYC amplifications
	BRCA2 mutation	N/A	May predict PARP inhibitor response
	ATM mutation	N/A	Potential role in DNA damage repair–targeted therapies
Kohli et al. 2020	AR amplification and SNVs (T742L, T742C, V716M, T878A, L702H, H875Y)	poor survival	Castration resistance and shorter survival on AR-targeted therapy
	TP53 missense mutations and deletions	Shorter OS in both mHSPC & mCRPC	Indicates aggressive phenotype; worse outcome on ADT
	RB1 copy number loss (homozygous/heterozygous deletion)	Independent marker of poor OS in mCRPC	Independent marker of poor prognosis
	ATM deleterious mutations	Shorter OS	worse response to ADT in mHSPC' Suggests impaired DNA repair; adverse on hormonal therapy
	BRCA1 deleterious mutation	Shorter OS	worse response to ADT in mHSPC; Indicates potential benefit from PARP inhibitors but poorer initial ADT response
	BRCA2 deleterious mutation	Shorter OS	worse response to ADT in mHSPC; Similar to BRCA1 effects
	CHEK2 deleterious mutation	Shorter OS	worse response to ADT in mHSPC; Impaired checkpoint function; adverse prognosis
	CDK6 gene amplification	Poor OS in mCRPC	May drive cell-cycle progression; potential target
	CDH1 deletion	Poor OS in mCRPC	Loss of adhesion; aggressive phenotype
	EGFR gene amplification	no clear survival link	Possible role in aggressiveness but not prognostic here
	MYC gene amplification	no clear survival link	Common event; prognostic impact unclear in this cohort
	BRAF gene amplification	no clear survival link	Limited prevalence; not independently prognostic
Torquato et al. 2019	AR ligand-binding domain mutation	shorter PFS	Associated with resistance to enzalutamide/abiraterone
	AR copy number (CN) gain	Worse PFS & OS in univariate; lost significance in multivariable	Suggests AR-driven resistance, though other factors may mediate outcome
	TP53 mutations	Strong association with worse OS, especially with RB1 loss	Indicates aggressive, treatment-resistant disease
	RB1 mutations	N/A for OS alone but linked to neuroendocrine transformation & AR resistance	Drives lineage plasticity and poor response
	PTEN deletion(s) and/or mutation(s)	N/A for direct OS/PFS here but linked to castration resistance	Associated with castration resistance and poor response to AR inhibitors

	PIK3CA copy number gain and/or mutation	worse OS	PI3K pathway defects confer poorer survival
	APC mutation(s)	Worse OS in univariate; not independent in multivariable	WNT pathway defects; prognostic only in unadjusted analysis
	BRCA1/2, ATM pathogenic mutations	Not significantly associated with worse outcomes in this study	In this cohort, no clear impact on survival
Jayaram et al. 2021	TP53 mutation and deletion	significantly shorter OS (HR: 7.13, $p < 0.001$) and PFS (HR: 2.69, $p = 0.035$).	Strong adverse prognostic marker; early predictor of poor abiraterone response
	RB1 deletion	shorter OS (HR: 6.24, $p = 0.002$).	Indicates aggressive disease; poor prognosis
	PTEN mutation and deletion	worst OS (HR: 11.9, $p < 0.001$).	Marker of highly aggressive, treatment-resistant phenotype
	AR copy number gain	Persisted at progression; de novo AR mutations emerged in 16%	Pre-treatment gain predicts resistance; emergence of mutations indicates adaptive resistance
	PIK3CA mutation and copy number gain	shorter OS ($p < 0.0001$)	PI3K pathway activation linked to poor survival
	DDR alterations (BRCA2, ATM, CHEK2, FANCA, HDAC2)	no impact on OS<br>significantly associated with shorter PFS (HR: 2.13, $p = 0.003$)	May predict earlier progression; unclear impact on overall survival without PARP therapy
De Laere et al. 2019	TP53 Mutation, Deletion	worse OS in mCRPC, (median: 7.8 vs. 26.7 mo, $P < 0.0001$).<br>worse and shorter PFS in mCRPC (median: 3.0 vs. 8.7 mo, $P < 0.0001$)	Strongest prognostic marker; outperforms AR biomarkers for predicting abiraterone/enzalutamide outcome
	AR Amplification, Structural Rearrangement	Associated with poor prognosis in univariate; not independent for PFS	Suggests AR-driven resistance but secondary to TP53 effect
	AR (L702H, H875Y)	not independently prognostic	May contribute to resistance mechanisms but overshadowed by tumor-suppressor status
Knutson et al. 2024	AR Copy number gain; Structural rearrangements (AR-GSRs) (and infrequent point mutations)	Poorer OS and rPFS	Indicates resistance to AR-targeted therapies; prognostic cfDNA marker
	TP53 Point mutations and copy number loss	Poorer survival outcomes	Adverse prognostic factor
	PTEN Copy number loss	Poor clinical outcomes	Aggressive disease marker
	RB1 Copy number loss	Worse prognosis	treatment resistance, indicates lineage plasticity; poor response
	MYC Copy number gain	poorer outcomes	contributes to ctDNA positivity ; Marker of aggressive biology
	MYCN Copy number gain	associated with adverse outcomes	Reflects neuroendocrine/AVPC features
Oya et al. 2022	HRR-pathway pathogenic genes (ATM, BRCA1, BRCA2, BARD1, BRIP1, CDK12, CHEK1, CHEK2, FANCL, PALB2, RAD51B, RAD51C, RAD51D, RAD54L): Pathogenic mutation	Improved rPFS with abiraterone + olaparib vs abiraterone alone (HR ~0.50; 95% CI 0.34–0.73).); trend to OS benefit (HR ~0.83, 95% CI 0.66–1.03)	HRR mutations predict greater benefit from adding olaparib to abiraterone in 1L mCRPC

	BRCA1/BRCA2: Pathogenic mutation	Greatest rPFS improvement (HR ~0.23; median NR vs 8.4 mo)	Strong indicator for combination therapy benefit
	Non-HRRm	rPFS benefit also observed (HR ~0.76)	Suggests some benefit beyond HRR-mutated subgroup, but magnitude less than HRRm
Annala et al. 2018	AR Amplifications	shorter TTP in univariate analyses	Indicates earlier progression on AR-targeted agents (enzalutamide/abiraterone)
	AR Ligand Binding Domain (LBD) Mutations (specifically W742C and H875Y)	Not explicitly linked to worse OS/PFS	responding clinically to enzalutamide and abiraterone; ; not clearly negative prognostic
	AR Genomic Structural Rearrangements (AR-GSRs)	Worse PFS	Predicts primary resistance to AR-targeted therapy; may prompt alternative strategies early
	HRR Defects (including somatic truncating mutations in BRCA2 and ATM)	Shorter PFS and OS	Poor response to AR-targeted therapy; may benefit from PARP inhibitors or platinum-based chemotherapy<br>Somatic LOH in BRCA2/ATM truncating mutations observed, indicating reliability in guiding therapy despite ctDNA levels
	TP53 Mutations/deletions	worse OS and PFS	Indicates risk of rapid progression, poor AR-targeted therapy response; closer monitoring indicated
	PI3K Pathway defects	PFS (inferable worse)	Associated with poor response to AR-targeted therapy; suggests combination trials with PI3K/AKT/mTOR inhibitors
Pan et al. (2022)	HRR mutation group (HRRmt): ATM, BRCA2, CDK12, CHEK2, BRIP1	significantly worse PFS on abiraterone (median 8.2 vs. 19.0 mo; $p = 0.002$)	Indicates intrinsic resistance to abiraterone; consider alternative or combination therapies (e.g., PARP inhibitors)
	PTEN loss of expression	no significant association with PFS (median 12.3 vs. 15.2 mo; $p = 0.952$).	Not predictive of abiraterone response/resistance in this cohort
Yuan et al. 2022	PALB2 c.751C>T (p.Q251*), Exon 4	N/A (case-report context)	Initial good response to olaparib; indicates PARP inhibitor sensitivity mutations
	PALB2 c.751_752delCAinsTT (p.Q251L), Exon 4<br>PALB2 c.751_753delCAGinsTAC (p.Q251Y), Exon 4	N/A	Somatic reversion mutations restoring HRR function → resistance to olaparib
	PTEN c.136_137del (p.Y46Qfs*5), Exon 2	Worse prognosis (inferred shorter PFS/OS)	Associated with resistance to ADT; suggests aggressive phenotype and need for alternative strategies

	AR Amplification	Worse PFS: median 3.9 vs 9.5 months, p < 0.0001; Worse OS: median 11.2 vs 29.0 months, p < 0.0001	Predicts resistance to abiraterone/enzalutamide; consider earlier switch or combination approaches
	CHD1 loss/deletion	worse metastasis-free survival (in broader context); role in therapy resistance not fully defined	Potential cooperation with SPOP mutations; may influence response patterns; further study needed
	FGFR1 amplification	Not significantly associated with OS: HR 2.34 (95% CI: 0.84–6.54), p = 0.11	Potential target for FGFR inhibitors (e.g., erdafitinib) but no clear prognostic link here
	TP 53 c.665_672*11del, Exon 6-IVS6	Worse OS: median 7.3 vs 26.7 months, p < 0.001; Worse PFS: median 3.3 vs 10.0 months, p < 0.0001	Poor response to AR-targeted therapy; indicates aggressive, treatment-resistant phenotype
	NOTCH2 c.5311-1G>A, IVS29	N/A	Clinical significance unclear; potential resistance roles not defined
	PIK3C2G c.2143A>G (p.R715G), Exon 15	N/A	Potential contribution to ADT/PARP inhibitor resistance
	LHCGR c.143C>T (p.T48M), Exon 1	N/A	Potential influence on hormonal signaling
	CDC25C c.1150_1151delGGinsCC (p.G384P), Exon 12	N/A	Potential disruption of DNA damage response
	FLT4 c.376G>A (p.A126T), Exon 3	N/A	Potential role in angiogenesis
Carr et al. 2021	BRCA2 Frameshift, nonsense, Copy number deletions	Better rPFS: HR 0.34; 95% CI 0.25–0.47; p < 0.001	Predicts strong benefit from olaparib in mCRPC; key biomarker for PARP inhibitor selection
	ATM Frameshift, Copy number deletions	Modest PFS benefit: HR 0.93 (95% CI 0.53–1.62); no significant OS benefit	Less pronounced benefit from olaparib; may require combination or alternative strategies
	CDK12 Point mutation	No significant rPFS benefit with olaparib (HR ~1.11 for non-BRCA HRR group)	Not clearly predictive of PARP inhibitor response; clinical role unclear
	CHEK2 Missense	No statistically significant PFS/OS improvement	Unclear predictive role; included in “other HRR” group with mixed outcomes
	PALB2 Missense	Limited clinical data; potential benefit not confirmed; mixed outcomes	May respond to PARP inhibitors but evidence limited; consider in context of other HRR alterations
	PPP2R2A Copy number loss	N/A	N/A
Du et al. 2023	CDK12 point mutation	TCR: 8.43 months vs. 17.70 months, p < 0.001	High mutation burden; sensitivity to immune checkpoint blockade
	AR point mutation	N/A	N/A
	NCOR2 point mutation	N/A	N/A
	ATR point mutation	N/A	N/A
	BRCA2 point mutation	TCR: 8.02 vs. 13.20 months, p = 0.011	Associated with worse clinical outcomes; ; may guide PARP inhibitor consideration

	ATM point mutation	TCR: 8.02 vs. 13.20 months, p = 0.011	Associated with worse clinical outcomes; ; potential for DDR-targeted therapies
	Germline BRCA2 point mutation	N/A	N/A
	Baseline ctDNA positivity (any mutation)	Worse rPFS, Baseline ctDNA positivity: HR = 1.8, p < 0.01; Multivariate Cox model: HR = 1.6, p = 0.011	Identifies poorer prognosis at baseline; may guide intensification or closer monitoring
Goodall et al., 2020	Changes in ctDNA at C3D1 (reduction vs increase)	Superior rPFS with reduction (HR 2.0 for increase vs reduction; p<0.01)	Early on-treatment ctDNA drop predicts response (CR/PR/SD vs PD) and rPFS
	TP53, AR, FOXA, PTEN, PI3K/AKT	N/A	Emerging resistance mutation Guides identification of resistance mechanisms; may inform next-line therapy choices (e.g., switch to non-AR therapies)
Kristiansen et al. 2024	TP53 Mutation (inactivation/loss-of-function)	Inferior PFS :STR 0.76 (90% CI: 0.57–1.05), and inferior OS	Suggests poorer outcomes on platinum; may indicate need for alternative treatments beyond carboplatin
	DRD including (BRCA1/2) Mutation/inactivation	Inferior PFS :STR 0.68 (90% CI: 0.45–1.07), and inferior OS	Despite hypothesized platinum sensitivity, carboplatin was not favorable; may need different DDR-targeting strategies
	AR Alteration	Shorter median rPFS: 5.0 vs 11.6 months (HR 1.954, p<0.001)	Associated with poorer rPFS on ARPI; but may predict greater benefit from 177Lu-PSMA-617 vs ARPI change
De Bono et al. 2024	TP53 Alteration	Shorter median rPFS: 6.1 vs 9.2 months (HR 1.655, p<0.01)	Similar pattern: worse on ARPI but improved rPFS when switching to 177Lu-PSMA-617
	PTEN Alteration	Shorter median rPFS: 3.6 vs 7.9 months (HR 1.62, p<0.05)	Indicates poor ARPI response; 177Lu-PSMA-617 may overcome resistance
